# Cortical lipid metabolic pathway alteration of early Alzheimer’s disease and candidate drugs screen

**DOI:** 10.1186/s40001-024-01730-w

**Published:** 2024-03-25

**Authors:** Linshuang Wang, Fengxue Qu, Xueyun Yu, Sixia Yang, Binbin Zhao, Yaojing Chen, Pengbo Li, Zhanjun Zhang, Junying Zhang, Xuejie Han, Dongfeng Wei

**Affiliations:** 1 Institute of Basic Research in Clinical Medicine, China Academy of Chinese Medical Sciences, Beijing, 100700 China; 2 Beijing Anzhen Hospital, Capital Medical University, Beijing, 100029 China; 3 School of Traditional Chinese Medicine, Southern Medical University, Guangzhou, 510515 China; 4 State Key Laboratory of Cognitive Neuroscience and Learning & IDG/McGovern Institute for Brain Research, Beijing Normal University, Beijing, 100875 China; 5 BABRI Centre, Beijing Normal University, Beijing, 100875 China; 6 Institute of Gerontology, Hubei University of Chinese Medicine, Wuhan, 430065 China

**Keywords:** Alzheimer’s disease, Early-onset, Cortex, Lipid metabolism disorder, Bioinformatics, Candidate compounds

## Abstract

**Background:**

Lipid metabolism changes occur in early Alzheimer's disease (AD) patients. Yet little is known about metabolic gene changes in early AD cortex.

**Methods:**

The lipid metabolic genes selected from two datasets (GSE39420 and GSE118553) were analyzed with enrichment analysis. Protein–protein interaction network construction and correlation analyses were used to screen core genes. Literature analysis and molecular docking were applied to explore potential therapeutic drugs.

**Results:**

60 lipid metabolic genes differentially expressed in early AD patients’ cortex were screened. Bioinformatics analyses revealed that up-regulated genes were mainly focused on mitochondrial fatty acid oxidation and mediating the activation of long-chain fatty acids, phosphoproteins, and cholesterol metabolism. Down-regulated genes were mainly focused on lipid transport, carboxylic acid metabolic process, and neuron apoptotic process. Literature reviews and molecular docking results indicated that ACSL1, ACSBG2, ACAA2, FABP3, ALDH5A1, and FFAR4 were core targets for lipid metabolism disorder and had a high binding affinity with compounds including adenosine phosphate, oxidized Photinus luciferin, BMS-488043, and candidate therapeutic drugs especially bisphenol A, benzo(a)pyrene, ethinyl estradiol.

**Conclusions:**

AD cortical lipid metabolism disorder was associated with the dysregulation of the PPAR signaling pathway, glycerophospholipid metabolism, adipocytokine signaling pathway, fatty acid biosynthesis, fatty acid degradation, ferroptosis, biosynthesis of unsaturated fatty acids, and fatty acid elongation. Candidate drugs including bisphenol A, benzo(a)pyrene, ethinyl estradiol, and active compounds including adenosine phosphate, oxidized Photinus luciferin, and BMS-488043 have potential therapeutic effects on cortical lipid metabolism disorder of early AD.

**Supplementary Information:**

The online version contains supplementary material available at 10.1186/s40001-024-01730-w.

## Introduction

Alzheimer’s disease (AD) is the most prevalent form of dementia, with symptoms ranging from episodic memory impairments to severe cognitive decline and total reliance on caretakers [1]. According to the World Health Organization reports, the number of people living with AD has risen to 50 million, and by 2050, this figure is predicted to rise to 152 million [2]. Abnormal biomarkers in the pre-clinical stage of AD can help early diagnose AD [3]. However, since the overlap of early AD markers with normal aging [4, 5], the pathological mechanisms and potential targets of early AD remained resistant to analysis. Novel biomarkers for early detection of AD can aid in finding effective treatments [6, 7]. Lipid metabolism has gradually become an important research direction in early AD. Lipids play a crucial role in sustaining brain function. Both aging and early AD are accompanied by brain lipid metabolism disturbance. Recently, increasing research has shown that lipid metabolism played a role in early AD pathogenesis, and that changes in lipid metabolism may be a contributing factor to cognitive impairment [8]. Compared to healthy brains, the lipid profiles had been reported to be altered, and the concentrations of 420 lipid metabolites significantly differed in early AD brains [9].

Clinical studies have demonstrated significant alterations in lipid metabolism in early AD patients’ cortex. Different lipid species were depleted in both white and gray matter regions of the frontal cortex of early AD patients ranging from pre-clinical to severe stages. The content of glucosylceramide, sphingomyelin, and phosphatidylcholine decreased in white matter. In AD patients, the amount and activity of acid ceramidase were raised in the frontotemporal gray matter [10]. The composition of phospholipids and sulfatides was decreased by lipid composition analysis in tissue homogenates of brain samples from early AD patients [11, 12]. The protein levels of lipid mediators (LM) in the human entorhinal cortex (ENT) were measured, and resolvin D5 (RvD5), maresin 1 (MaR1), and protectin D1 (PD1) were much lower in AD patients, but prostaglandin D2 (PGD2) levels were higher [13].

In vitro experiments demonstrated significant alternations of lipid metabolism in rats and cell models. The lipidomic analysis revealed that sphingolipids and glycerophospholipids, such as Cer (ceramide), PE (phosphatidylethanolamine), LPE (lysophosphatidylethanolamine), and PC (phosphatidylcholine), were dysregulated in the brain of AD rats [14]. The dysregulated sphingolipid metabolism, glycerophospholipid metabolism, linoleic acid metabolism, and alpha-linolenic acid metabolism were found in the cerebral cortex of AD rats, by reducing pro-inflammatory gene expression (*IL-1β*, *TNF-α*) and promoting the production of anti-inflammatory genes (*Ym1*, *Fizz1*, *Arg1*, *TGF-β*). PPAR activation affects the inflammatory milieu of the brain, making microglia able to clear amyloid plaques [15]. However, precisely which changes of pathways are involved in AD pathology in response to aberrant lipid gene expression remains an open question. Thus, further studies are warranted to interrogate the potential effects of cortical lipid metabolic genes and related pathways in early AD.

The research utilizes microarrays technology to detect early AD cortical gene chip data, revealing key information related to AD onset. In comparison to traditional laboratory techniques, microarrays detection technology exhibits unique and robust features, characterized by high throughput, allowing simultaneous assessment of the expression levels of thousands of genes. This enables a comprehensive understanding of the differences between early-stage AD patients and health subjects. Through the application of this technology, we can uncover potential targets related to lipid metabolism, providing crucial clues for a deeper understanding of the molecular mechanisms underlying early-stage AD. By systematically analyzing rich gene expression data in public databases, we have identified genes closely associated with lipid metabolism disorder, further unveiling the potential biological foundations of AD onset.

Recently, integrated bioinformatics analysis has been widely used to explore the pathogenesis of multi-factorial diseases. Gene Ontology (GO) function analysis, Kyoto Encyclopedia of Genes and Genomes (KEGG) pathway enrichment analysis, and protein–protein interaction (PPI) networks are crucial for interpreting large-scale microarray data. These methods may extract relevant biological information from a wide range of gene set collections, providing vital new insights into disease mechanisms at the molecular level [15]. Molecular docking was used to verify the association between candidate compounds and core targets. In this study, experimental protein structures obtained from PDB database and predicted structures from AlphaFold database were combined to predict protein–ligand interactions. The Protein Data Bank (PDB) is the single global archive of the experimentally determined 3D structures of biological molecules [16]. AlphaFold is an AI system that has good accuracy in reproducing protein topology and binding site [17]. As for proteins that have no corresponding structure in the PDB, AlphaFold can make accurate predictions from the amino acid sequence [18].

Several biological processes have been demonstrated to correlate with early AD, including energy metabolism, cholesterol synthesis, calcium signaling, and biological pathways related to synaptic function and neurotransmission [7, 19]. However, the exact relationships and interactions of altered lipid metabolic genes remain unclear in the early AD cortex. Therefore, screening and identifying useful lipid metabolism biomarkers were necessary for early AD diagnosis and developing treatment strategy and assessment of patient prognosis.

Our study aims to identify core targets for early AD lipid metabolism using bioinformatics analyses and screen candidate therapeutic drugs by literature analysis and molecular docking. This study provides a scientific basis for diagnosing and treating early AD.

## Materials and methods

### Data source

The GSE39420 and GSE118553 gene expression profile datasets were obtained from the GEO database (https://www.ncbi.nlm.nih.gov/geo/), which is an open functional genomics database of high-throughput resources and includes gene expression, microarray, and chips data [20].

### Screen differential lipid metabolic genes

The GSE118553 and GSE39420 datasets were analyzed, respectively. These differentially expressed genes with fold change (FC) > 1.2 and *p* value < 0.05 were considered significant. Differentially expressed genes were then uploaded to UniProt (https://www.uniprot.org/uniprot/) and selected according to their known functions one by one. Differential lipid metabolic genes were ultimately screened.

### Enrichment analysis

Many metabolic, regulatory, and signal transduction processes are involved in the cortex. The differentially expressed genes were identified by GO enrichment analysis. PANTHER tools (version 16.0, www.pantherdb.org) with high reliability were performed to classify the molecular functions, biological processes, cellular components, protein classifications, and pathways.

### Network construction

High-quality regulated PPI networks were built using STRING analysis to further understand gene interactions. The list of 30 up-regulated and 30 down-regulated gene IDs was input into the STRING 11.0 database to identify known and predicted gene functional association networks. The PPI network was visualized using Cytoscape software (version 3.8.2). The subnetworks were constructed by CytoHubba [21] based on the interaction of network nodes.

### Explored the relevance of core genes to neurodegenerative diseases

The Comparative Toxicogenomics Database (CTD, http://ctdbase.org/) [22] was used to investigate the connections between core genes and neurodegenerative diseases and early AD-related symptoms.

### Corresponding compounds and candidate drugs screen

The efficient therapeutic drugs targeting the core pathogenic genes were screened for further investigations. The potential compounds targeting these core targets were collected from literature and the DrugBank database (DrugBank, https://www.drugbank.ca/). The candidate drugs that can regulate core gene expression were screened by CTD. A literature review was conducted to discover the regulations between screened core genes and neurodegenerative diseases, especially early AD.

### Molecular docking

The chemical structures of compounds were obtained from the PubChem database (https://pubchem.ncbi.nlm.nih.gov/). The structures of hub proteins were accessed from the RCSB Protein Data Bank (http://www.rcsb.org/) and AlphaFold Protein Structure Database (https://alphafold.ebi.ac.uk/). The docking studies of the core targets and candidate compounds were performed using AutoDock Tools 4.2 and AutoDock Vina (version 1.1.2) to calculate the binding energy. The docking results were visualized using PyMol software (version 2.2.0). The lowest binding energy score is considered to be the best solution.

### Statistical analysis

All values are shown as the mean ± standard deviation (SD). Statistical analyses were performed using SPSS20.0. The Demographic data were analyzed with an independent-sample *t*-test between the control group and the early AD group. *P* values < 0.05 were considered statistically significant.

## Results

### Screened genes related to cortical lipid metabolism

**Fig. 1:**
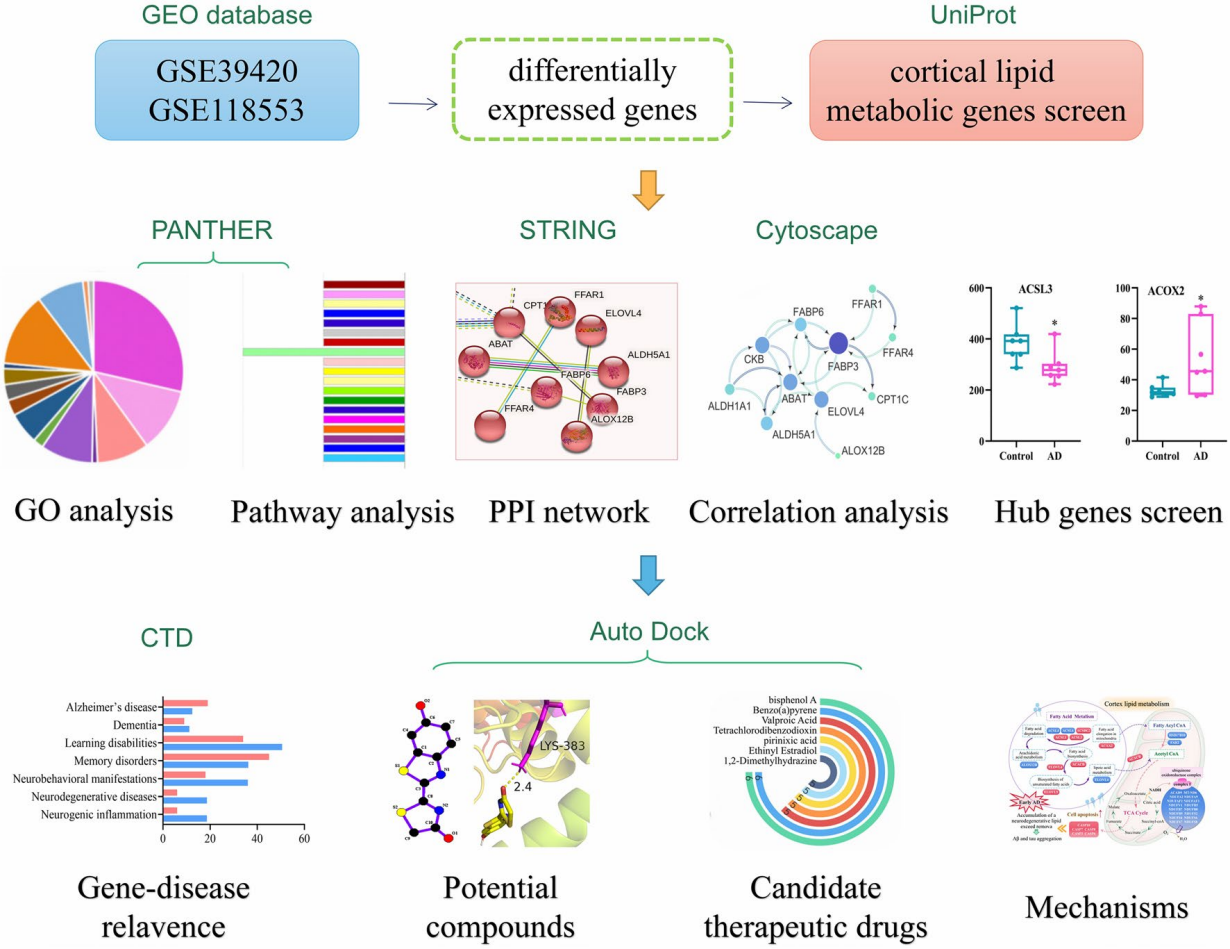
Schematic diagram of this study

The schematic diagram of the study is shown in Fig. 1. Data for 47 early AD patients and 34 healthy controls were screened (Table 1). The GSE3942 dataset contains 3 specimens (7 controls, 7 early-onset Alzheimer’s disease, 7 early-onset AD genetically determined by a mutation in *PSEN1* gene). GSE118553 dataset contains 3 specimens (27 controls, 33 AsymAD, and 52 AD patients). Specimens in two GEO datasets were classified as two groups (the control group and the early AD group). In total, 30 up-regulated and 30 down-regulated genes associated to lipid metabolism were selected in our study. DEGs were simultaneously identified by the two algorithms. The screening results for 30 down-regulated lipid metabolism-associated genes are shown in Table 2, and the molecular function and biological process category of these genes are shown in Additional file 1: Table S1. The screening results for 30 up-regulated lipid metabolism-associated genes are shown in Table 3, and the molecular function and biological process category of these genes are shown in Additional file 1: Table S2.

**Table 1:** Summary of AD microarray datasets from GEO datasets

No.	Series	Samples	Source	Platform	Affymetrix GeneChip
1	GSE39420	7 controls, 7 early-onset Alzheimer’s disease,7 early-onset AD genetically determined by a mutation in PSEN1 gene	The posterior cingulate area	GPL11532	[HuGene-1_1-st] Affymetrix Human Gene 1.1 ST Array
2	GSE118553	27 controls, 33 AsymAD, and 52 AD subjects	Entorhinal cortex, temporal cortex, frontal cortex, cerebellum	GPL10558	Illumina HumanHT-12 V4.0 expression beadchip

**Table 2:** The screen results of 30 down-regulated lipid metabolism-associated genes

No.	Accession no.	Gene symbol	Gene name	*P* value	FC (AD/control)
1	8082444	*ACAD9*	Acyl-CoA dehydrogenase family member 9	0.017	0.747
2	ILMN_1671633	*ACOT4*	Acyl-CoA thioesterase 4	0.000	0.703
3	7912012	*ACOT7*	Acyl-CoA thioesterase 7	0.011	0.707
4	8066598	*ACOT8*	Acyl-coenzyme A thioesterase 8	0.002	0.747
5	8174474	*ACSL4*	Long-chain-fatty-acid–CoA ligase 4	0.016	0.713
6	8113938	*ACSL6*	Acyl-CoA synthetase long-chain family member 6	0.045	0.566
7	ILMN_1661596	*ALDH1A1*	Aldehyde dehydrogenase 1 family member A1	0.041	0.803
8	8090314	*ALDH1L1*	Aldehyde dehydrogenase 1 family member L1	0.045	0.736
9	8117207	*ALDH5A1*	Aldehyde dehydrogenase 5 family member A1	0.014	0.668
10	8012309	*ALOX12B*	Arachidonate 12-lipoxygenase, 12R type	0.000	0.673
11	7944418	*C2CD2L*	Phospholipid transfer protein C2CD2L	0.040	0.773
12	7938090	*CKB*	Creatine kinase B-type	0.011	0.315
13	8030448	*CPT1C*	Carnitine palmitoyltransferase 1C	0.018	0.618
14	8164535	*CRAT*	Carnitine *O*-acetyltransferase	0.026	0.781
15	8127767	*ELOVL4*	Elongation of very long-chain fatty acids protein 4	0.032	0.425
16	7914342	*FABP3*	Fatty acid-binding protein 3	0.047	0.443
17	8109629	*FABP6*	Fatty acid-binding protein 6	0.024	0.582
18	8018251	*FADS6*	Fatty acid desaturase 6	0.040	0.713
19	7954631	*FAR2*	Fatty acyl-CoA reductase 2	0.025	0.444
20	7943075	*FAT3*	FAT atypical cadherin 3	0.022	0.446
21	7929344	*FFAR4*	Free fatty acid receptor 4	0.025	0.496
22	ILMN_1658548	*HSD17B10*	3-Hydroxyacyl-CoA dehydrogenase type-2	0.001	0.808
23	8020308	*LDLRAD4*	Low-density lipoprotein receptor class A domain containing 4	0.036	0.828
24	8085984	*OSBPL10*	Oxysterol-binding protein like 10	0.024	0.546
25	7897089	*PLCH2*	Phospholipase C eta 2	0.031	0.755
26	8078187	*PLCL2*	Phospholipase C like 2	0.012	0.470
27	ILMN_1654653	*PLCXD2*	Phosphatidylinositol specific phospholipase C X domain containing 2	0.002	0.677
28	8028791	*PLD3*	Phospholipase D family member 3	0.012	0.595
29	8025877	*PLPPR2*	Phospholipid phosphatase related 2	0.033	0.734
30	7917946	*PLPPR5*	Phospholipid phosphatase related 5	0.019	0.366

*FC* fold change

**Table 3:** The screen results of 30 up-regulated lipid metabolism-associated genes

No.	Accession no.	Gene symbol	Gene name	*P* value	FC (AD/control)
1	8023261	*ACAA2*	Acetyl-CoA acyltransferase 2	0.015	1.370
2	ILMN_1671557	*ACACB*	Acetyl-CoA carboxylase beta	0.000	1.537
3	8088397	*ACOX2*	Acyl-CoA oxidase 2	0.037	1.643
4	8025011	*ACSBG2*	Acyl-CoA synthetase bubblegum family member 2	0.025	1.202
5	8103951	*ACSL1*	Long-chain-fatty-acid–CoA ligase 1	0.003	1.976
6	ILMN_1651699	*ACSL3*	Long-chain-fatty-acid–CoA ligase 3	0.024	1.227
7	8029536	*APOC1*	Apolipoprotein C1	0.018	2.013
8	ILMN_1671935	*APOC4*	Apolipoprotein C4	0.029	1.365
9	8075695	*APOL3*	Apolipoprotein L3	0.036	1.203
10	8075709	*APOL4*	Apolipoprotein L4	0.047	1.763
11	7954036	*APOLD1*	Apolipoprotein L domain containing 1	0.005	1.417
12	7949916	*CHKA*	Choline kinase alpha	0.014	1.435
13	8127145	*ELOVL5*	ELOVL fatty acid elongase 5	0.003	1.376
14	7929032	*FAS*	Fatty acid synthase	0.012	1.713
15	8104079	*FAT1*	FAT atypical cadherin 1	0.003	1.765
16	8049825	*HDLBP*	High-density lipoprotein-binding protein	0.011	1.532
17	8025828	*LDLR*	Low-density lipoprotein receptor	0.004	2.798
18	7939376	*LDLRAD3*	Low-density lipoprotein receptor class A domain containing 3	0.013	1.636
19	ILMN_1671500	*OSBPL6*	Oxysterol-binding protein like 6	0.000	11.834
20	7908351	*PLA2G4A*	Phospholipase A2 group IVA	0.049	1.417
21	7929388	*PLCE1*	Phospholipase C epsilon 1	0.005	2.509
22	8167449	*PLP2*	Proteolipid protein 2	0.035	1.822
23	8031999	*PLPP2*	Phospholipid phosphatase 2	0.020	1.752
24	7930980	*PLPP4*	Phospholipid phosphatase 4	0.039	1.815
25	8091327	*PLSCR1*	Phospholipid scramblase 1	0.010	2.011
26	8091306	*PLSCR4*	Phospholipid scramblase 4	0.033	1.745
27	8066619	*PLTP*	Phospholipid transfer protein	0.011	1.996
28	7907702	*SOAT1*	Sterol *O*-acyltransferase 1	0.000	1.510
29	7970924	*STARD13*	StAR-related lipid transfer domain containing 13	0.030	1.290
30	8053890	*STARD7*	StAR-related lipid transfer domain containing 7	0.000	1.502

### GO analysis results of down-regulated genes

**Fig. 2:**
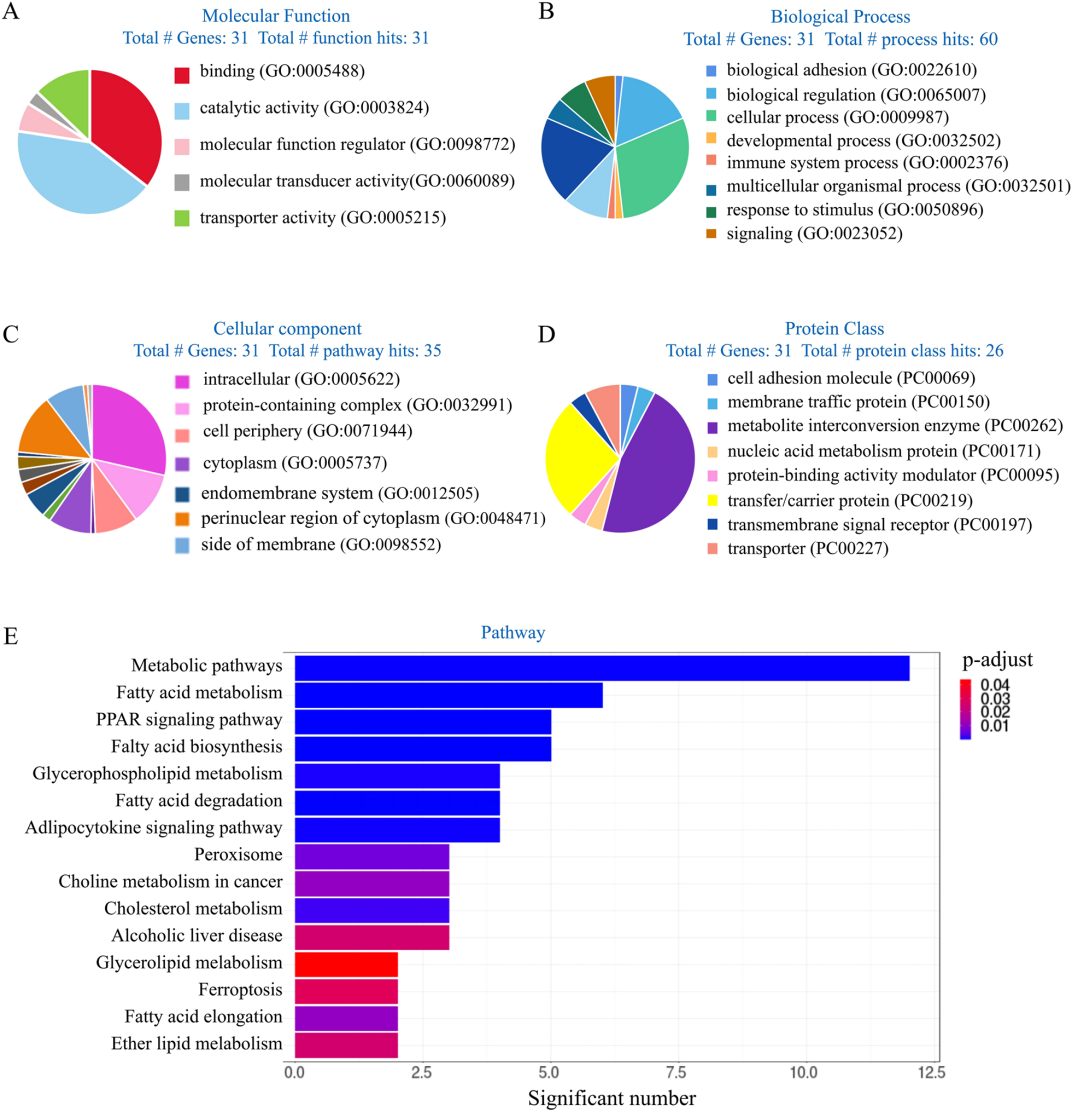
Functional enrichment analysis of the 30 down-regulated genes. **a** Molecular function results of down-regulated genes. **b** Biological process results of down-regulated genes. **c** Cellular component results of down-regulated genes. **d** Protein class results of down-regulated genes. **e** Pathway analysis of down-regulated genes

GO analysis was performed to classify the down-regulated genes into biological process, cellular component, protein class, and pathways. The results showed that down-regulated genes were involved in lipid metabolism. Molecular function results indicated that 73.1% of these 31 genes were related to catalytic activity. 36.8%, 10.5%, 31.6%, and 21.1% of this category were hydrolase activity, ligase activity, oxidoreductase activity, and transferase activity, respectively (Fig. 2a). Most of the genes were determined to be involved in the cellular process (38.3%) and metabolic process (29.8%) in biological processes hits. Biological adhesion, multicellular organismal process, and developmental process each account for 2.1% of all genes. Signaling, response to stimulus, and biological regulation account for 8.5%, respectively (Fig. 2b). Cellular component results indicated that most genes were involved in the intracellular organelle (22.9%), cytoplasm (25.1%), and organelle (12.2%) (Fig. 2(c)). In protein classification, 91.3% of genes were localized in metabolite interconversion enzyme, involving hydrolase (34.8%), ligase (8.7%), oxidoreductase (26.1%), and transferase (21.7%). Others were localized in cell adhesion molecule and transmembrane signal receptor, each with 4.3% (Fig. 2d). According to the classification of pathways, PPAR signaling pathway, adipocytokine signaling, glycerophospholipid metabolism, pathway, fatty acid degradation, fatty acid biosynthesis, ferroptosis, fatty acid elongation, and biosynthesis of unsaturated fatty acids were essential signaling pathways involved in the cortical lipid metabolism (Fig. 2e).

### GO analysis results of up-regulation genes

**Fig. 3:**
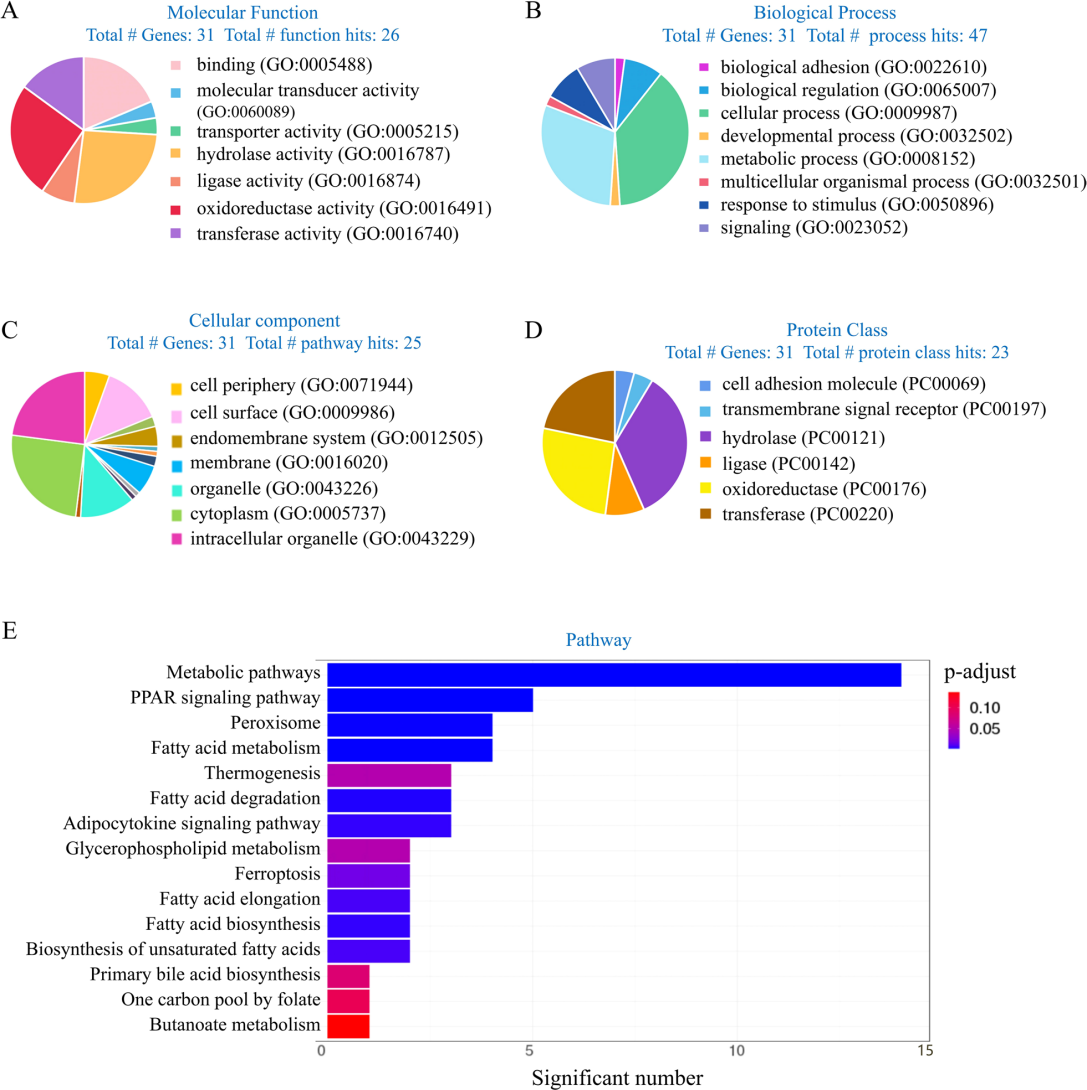
Functional enrichment analysis of the 30 up-regulated genes. **a** Molecular function results of up-regulated genes. **b** Biological process results of up-regulated genes. **c** Cellular component results of up-regulated genes. **d** Protein class results of up-regulated genes. **e** Pathway analysis of up-regulated genes

GO analysis results of up-regulated genes involved in lipid metabolism are shown in Fig. 3. Molecular function results indicated that 35.5%, 41.9%, 6.5%, 3.2%, and 12.9% of these 31 proteins were binding, catalytic activity, molecular function regulator molecular transducer activity, and transporter activity, respectively (Fig. 3a). In biological process classification, most of the genes were determined to be involved in cellular process (30.0%) and metabolic process (20.0%). Others were related to biological regulation (16.7%), localization (10.0%), etc. (Fig. 3b). Cellular component results indicated that most genes were involved in intracellular (28.7%), membrane (13.1%), and protein-containing complex (11.4%) (Fig. 3c). In protein class, 46.2% of genes were localized in cell part, 26.9% in transfer/carrier protein, and others were localized to transporter (7.7%). (Fig. 3d). According to the classification of pathways, PPAR signaling pathway, fatty acid biosynthesis, glycerophospholipid metabolism, fatty acid degradation, adipocytokine signaling pathway, and ferroptosis were essential signaling pathways involved in the cortical lipid metabolism (Fig. 3e).

### Functions classification of down-regulated genes

As shown in Table 4, early AD influenced seven specific molecular functions of cortical lipid metabolism, including binding, hydrolase activity, ligase activity, oxidoreductase activity, transferase activity, molecular transducer activity, and transporter activity. Among this classification, ACAD9, ACOT7, and FFAR4 occurred as repeats. Biological process included eight specific categories, mainly focusing on cellular process, metabolic process, etc. The results of gene classes mainly included hydrolase, oxidoreductase, and transferase. Among this classification, LDLRAD4 repeat appeared 12 times, with ACSL4, FFAR4, PLCH2, and PLCL2 appearing 4 times, respectively. The cellular component involved ten specific categories, including organelle, cytoplasm, and intracellular organelle. Among this classification, ACOT7 and ELOVL4 repeat appeared 12 times. The protein class included ten specific categories, mainly focusing on hydrolase and oxidoreductase. Among this classification, every gene is included only once. Among biological processes, 18 genes were involved in the cellular process, with 14 involved in the metabolic process. Gene classification results were focused on hydrolase activity and oxidoreductase activity. In pathway categories, 3 genes were involved in 5-Hydroxytryptamine degradation. Pathway analysis results mainly focus on metabolic pathways, PPAR signaling pathway, and peroxisome. Among this classification, ACSL1, ACSL3, PLTP, and ACOX2 are hub proteins that appear multiple times in significantly enriched pathways. Overall, ACOT7, ACSL4, and LDLRAD4 proteins occurred with a maximum frequency, closely followed by ELOVL4, which also has the high frequencies of most functional classes.

**Table 4:** The GO function classes of 30 down-regulated genes related to lipid metabolism

No.	Category	Gene symbol
*Molecular function*		
1	Binding (GO:0005488)	OSBPL10, ACOT7, FFAR4, ACAD9, LDLRAD4
2	Hydrolase activity (GO:0016787)	ACOT4, ACOT7, PLPPR5, PLPPR2, PLCL2, ACOT8, PLCH2
3	Ligase activity (GO:0016874)	ACSL6, ACSL4
4	Oxidoreductase activity (GO:0016491)	ALDH5A1, ALDH1L1, ACAD9, ALOX12B, FAR2, ALDH1A1
5	Transferase activity (GO:0016740)	CRAT, CKB, CPT1C, ELOVL4
6	Molecular transducer activity (GO:0060089)	FFAR4
7	Transporter activity (GO:0005215)	OSBPL10
*Biological process*		
1	Biological adhesion (GO:0022610)	FAT3
2	Biological regulation (GO:0065007)	FFAR4, PLCL2, PLCH2, LDLRAD4
3	Cellular process (GO:0009987)	FAT3, CRAT, ACOT4, ACOT7, FFAR4, CKB, PLPPR5, CPT1C, ALOX12B, PLPPR2, PLCL2, ACOT8, FAR2, ACSL6, PLCH2, ELOVL4, ACSL4, LDLRAD4
4	Developmental process (GO:0032502)	ACSL4
5	Metabolic process (GO:0008152)	CRAT, ACOT4, ACOT7, CKB, PLPPR5, CPT1C, ALOX12B, PLPPR2, ACOT8, FAR2, ACSL6, ELOVL4, ACSL4, LDLRAD4
6	Multicellular organismal process (GO:0032501)	ACSL4
7	Response to stimulus (GO:0050896)	FFAR4, PLCL2, PLCH2, LDLRAD4
8	Signaling (GO:0023052)	FFAR4, PLCL2, PLCH2, LDLRAD4
*Cellular component*		
1	Cell periphery (GO:0071944)	FFAR4, ACSL6, ELOVL4, ACSL4, LDLRAD4
2	Cell surface (GO:0009986)	FFAR4, ACSL6, ELOVL4, ACSL4, LDLRAD4
4	Cytosol (GO:0005829)	OSBPL10, ACOT7
5	Endomembrane system (GO:0012505)	ACSL6, ELOVL4, ACSL4, LDLRAD4
6	Extracellular region (GO:0005576)	ACOT7
7	Extracellular space (GO:0005615)	ACOT7
8	Intrinsic component of membrane (GO:0031224)	FFAR4, ELOVL4
9	Membrane (GO:0016020)	OSBPL10, FFAR4, ACSL6, ELOVL4, ACSL4, LDLRAD4
10	Membrane-enclosed lumen (GO:0031974)	ACOT7
11	Nucleoplasm (GO:0005654)	ACOT7
12	Organelle (GO:0043226)	CRAT, OSBPL10, ACOT4, ACOT7, ACAD9, CPT1C, FAR2, ACSL6, ELOVL4, ACSL4, LDLRAD4
13	Perinuclear region of cytoplasm (GO:0048471)	ALOX12B
14	Cytoplasm (GO:0005737)	CRAT, OSBPL10, ACOT4, ACOT7, ACAD9, CPT1C, ALOX12B, FAR2, ACSL6, ELOVL4, ACSL4, LDLRAD4
15	Intracellular organelle (GO:0043229)	CRAT, OSBPL10, ACOT4, ACOT7, ACAD9, CPT1C, FAR2, ACSL6, ELOVL4, ACSL4, LDLRAD4
*Protein class*		
1	Cell adhesion molecule (PC00069)	FAT3
2	Hydrolase (PC00121)	ACOT4, ACOT7, PLPPR5, PLD3, PLPPR2, PLCL2, ACOT8, PLCH2
3	Ligase (PC00142)	ACSL6, ACSL4
4	Oxidoreductase (PC00176)	HSD17B10, ALDH5A1, ALDH1L1, ACAD9, ALOX12B, ALDH1A1
5	Transferase (PC00220)	CRAT, CKB, CPT1C, ELOVL4
6	Transmembrane signal receptor (PC00197)	FFAR4
*Pathway*		
1	5-Hydroxytryptamine degradation (P04372)	ALDH1L1, ALDH1A1
2	Gamma-aminobutyric acid synthesis (P04384)	ALDH5A1
3	Histamine H1 receptor mediated signaling pathway (P04385)	PLCL2
4	Wnt signaling pathway (P00057)	FAT3

### Functions classification of up-regulated genes

As shown in Tables 5, 6, the classification and function analysis of up-regulation genes were performed. Differentially up-regulated genes can be divided into 5 clusters: protein classification, biological process, cellular component, protein class, and pathway.

**Table 5:** The molecular function, biological process, and protein class of 30 up-regulated genes related to lipid metabolism

No.	Category	Gene symbol
*Protein class*		
1	Cell adhesion molecule (PC00069)	FAT1
2	Membrane traffic protein (PC00150)	PLP2
3	Metabolite interconversion enzyme (PC00262)	ACSBG2, SOAT1, ACSL1, ELOVL5, ACSL3, FAS, APOC1, PLA2G4A, PLPP2, ACOX2, PLPP4, ACAA2
4	Nucleic acid metabolism protein (PC00171)	HDLBP
5	Protein-binding activity modulator (PC00095)	STARD13
6	Transfer/carrier protein (PC00219)	APOC4, APOC1, LDLRAD3, LDLR, APOL3, APOLD1, APOL4
7	Transporter (PC00227)	PLSCR4, PLSCR1
*Biological process*		
1	Biological adhesion (GO:0022610)	FAT1
2	Biological regulation (GO:0065007)	PLCE1, APOC4, PLSCR4, FAS, APOC1, PLTP, PLSCR1, PLPP2, STARD13, ACOX2
3	Cellular process (GO:0009987)	ACSBG2, SOAT1, ACSL1, ELOVL5, ACSL3, PLCE1, PLSCR4, FAS, APOC1, PLTP, PLA2G4A, PLSCR1, PLPP2, STARD13, FAT1, ACOX2, PLPP4, ACAA2
6	Localization (GO:0051179)	SOAT1, APOC4, PLSCR4, APOC1, PLTP, PLSCR1
7	Metabolic process (GO:0008152)	ACSBG2, SOAT1, ACSL1, ELOVL5, ACSL3, FAS, APOC1, PLA2G4A, PLPP2, ACOX2, PLPP4, ACAA2
8	Multicellular organismal process (GO:0032501)	ACSL3, APOC1, PLTP
9	Response to stimulus (GO:0050896)	PLCE1, FAS, PLPP2, STARD13

**Table 6:** The cellular components and pathways classes of 30 up-regulated genes related to lipid metabolism

No.	Category	Gene symbol
*Molecular function*		
1	Binding (GO:0005488)	SOAT1, APOC1, FAS, PLTP, APOL3, APOLD1, PLA2G4A, OSBPL6, HDLBP, ACOX2, APOL4
2	Catalytic activity (GO:0003824)	ACSBG2, SOAT1, ACSL1, ELOVL5, ACSL3, PLCE1, APOC1, PLA2G4A, PLPP2, STARD13, ACOX2, PLPP4, ACAA2
3	Molecular function regulator (GO:0098772)	APOC1, STARD13
4	Transporter activity (GO:0005215)	PLSCR4, PLTP, OSBPL6, PLSCR1
*Cellular component*		
1	Cellular anatomical entity (GO:0110165)	ACSBG2, SOAT1, ACSL1, ELOVL5, ACSL3, PLSCR4, PLTP, APOL3, APOLD1, PLP2, PLA2G4A, OSBPL6, PLSCR1, PLPP2, ACOX2, ACAA2, APOL4
2	Intracellular (GO:0005622)	ACSBG2, SOAT1, ACSL1, ELOVL5, ACSL3, PLA2G4A, OSBPL6, HDLBP, ACOX2, ACAA2
3	Protein-containing complex (GO:0032991)	APOC4, FAS, APOC1, HDLBP
*Pathway*		
1	5HT2 type receptor mediated signaling pathway (P04374)	PLCE1
2	Androgen/estrogen/progesterone biosynthesis (P02727)	SOAT1
3	Apoptosis signaling pathway (P00006)	FAS
4	Inflammation mediated by chemokine and cytokine signaling pathway (P00031)	PLA2G4A
5	PDGF signaling pathway (P00047)	STARD13
6	Wnt signaling pathway (P00057)	FAT1

Early AD influenced five specific molecular functions, including binding, catalytic activity, molecular function regulator, molecular transducer activity, and transporter activity. APOC1 occurred three times in this classification, which had the highest frequency. The biological process included ten specific categories, mainly including biological adhesion biological regulation, and cellular process. FAS repeat appeared six times among this classification, with APOC1 and PLPP2 appearing five times. The results of protein class were found mainly include cell adhesion molecule, membrane traffic protein, and metabolite interconversion enzyme. Among this classification, APOC1 and FAS occurred as repeats. Among biological processes, the cellular process was impacted most substantially because approximately 18 genes were involved. Protein classification results indicated that 12 genes were involved in the metabolite interconversion enzyme. In pathway categories, PLA2G4A was determined to play an important role, and involved 6 pathways. The results of pathway indicated that metabolic pathways, fatty acid metabolism, and PPAR signaling pathway contained the most significant number of proteins. CPT1C, ACSL, and ACSL4 are hub proteins that appear multiple times in significantly enriched pathways. Overall, FAS gene occurred with a maximum frequency, closely followed by PLA2G4A, APOC1, and PLCE1, which also have the high frequencies of most functional classes.

### PPI networks analysis results of the down-regulated proteins

**Fig. 4:**
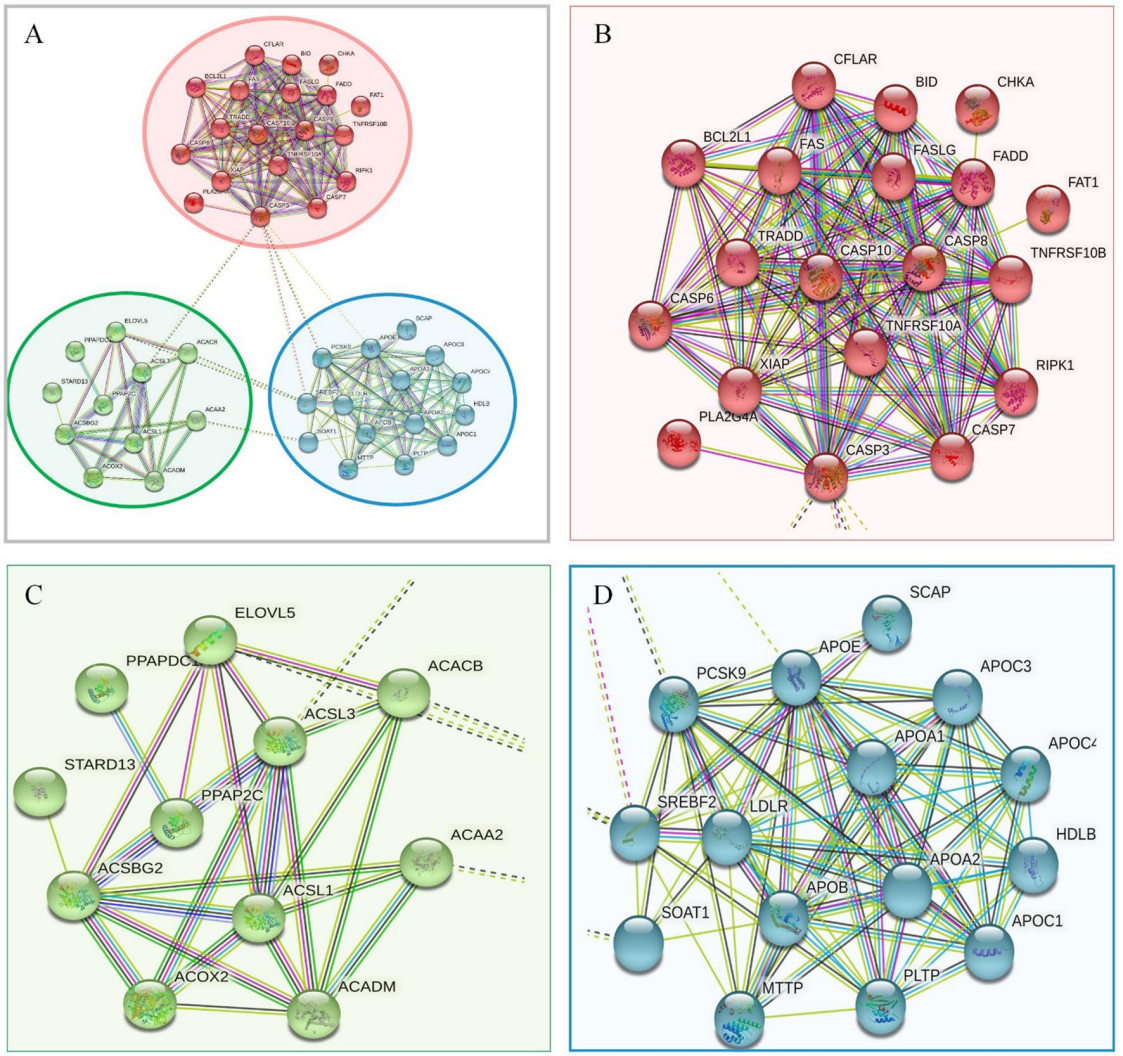
Classification based on biological functions of down-regulated proteins. Proteins in these clusters were mainly involved in neuron apoptotic process (blue), carboxylic acid metabolic process (green), lipid transport (red)

30 down-regulated proteins of lipid metabolism were uploaded for PPI network analysis. The PPI network consisted of 3 distinct function clusters, each of which contained identical function proteins and was represented by a different color (Fig. 4). 3 subnetworks consist of 30 down-regulated proteins that were related to regulating neuron apoptotic process (red), carboxylic acid metabolic process (green), and lipid transport (blue), respectively.

### PPI networks analysis results of the up-regulated proteins

**Fig. 5:**
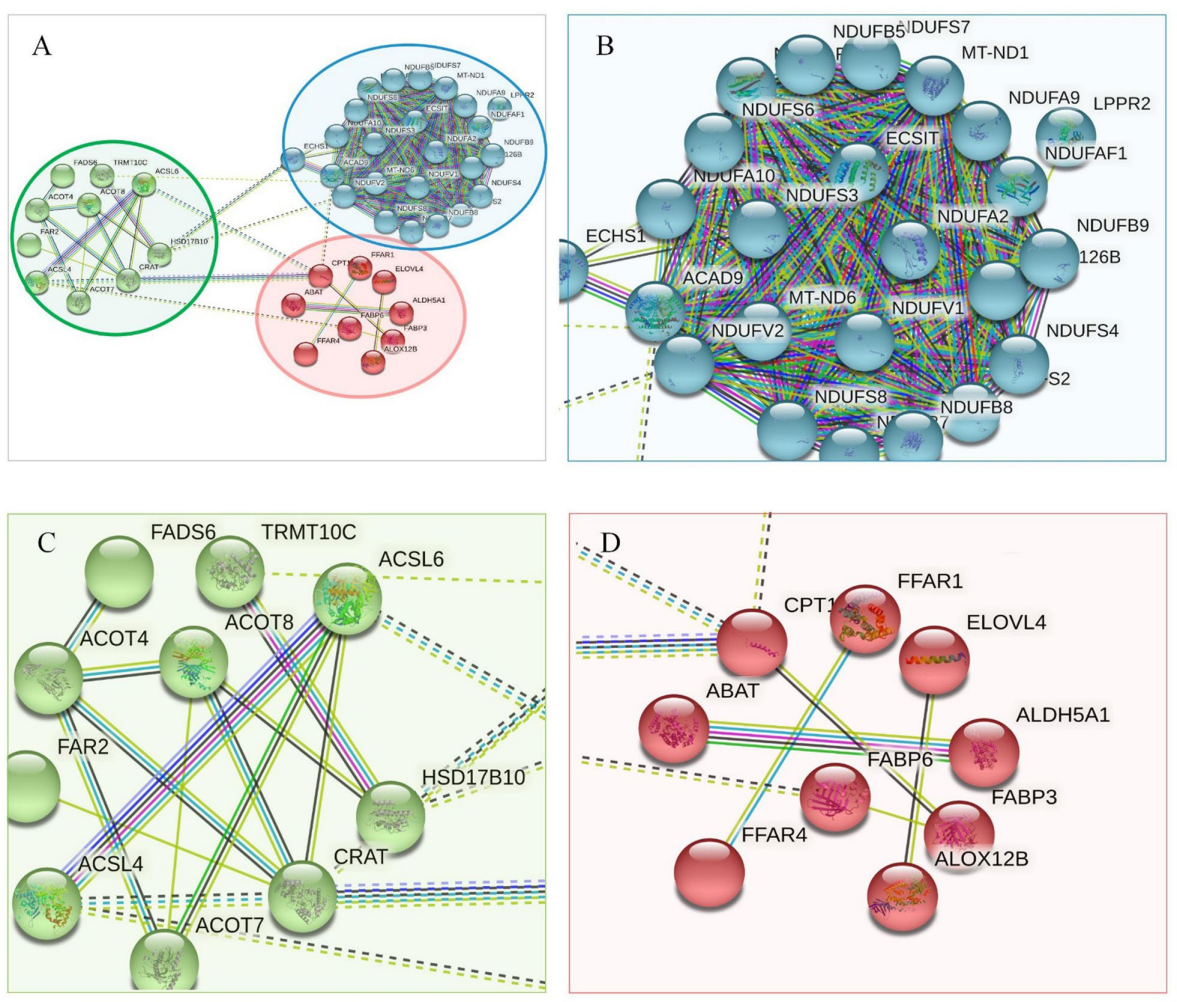
Classification based on biological functions of up-regulated proteins. Proteins in these clusters were mainly involved in mitochondrial fatty acid oxidation and long-chain fatty acids (green), phosphoprotein (red), cholesterol metabolism (blue)

**Fig. 6:**
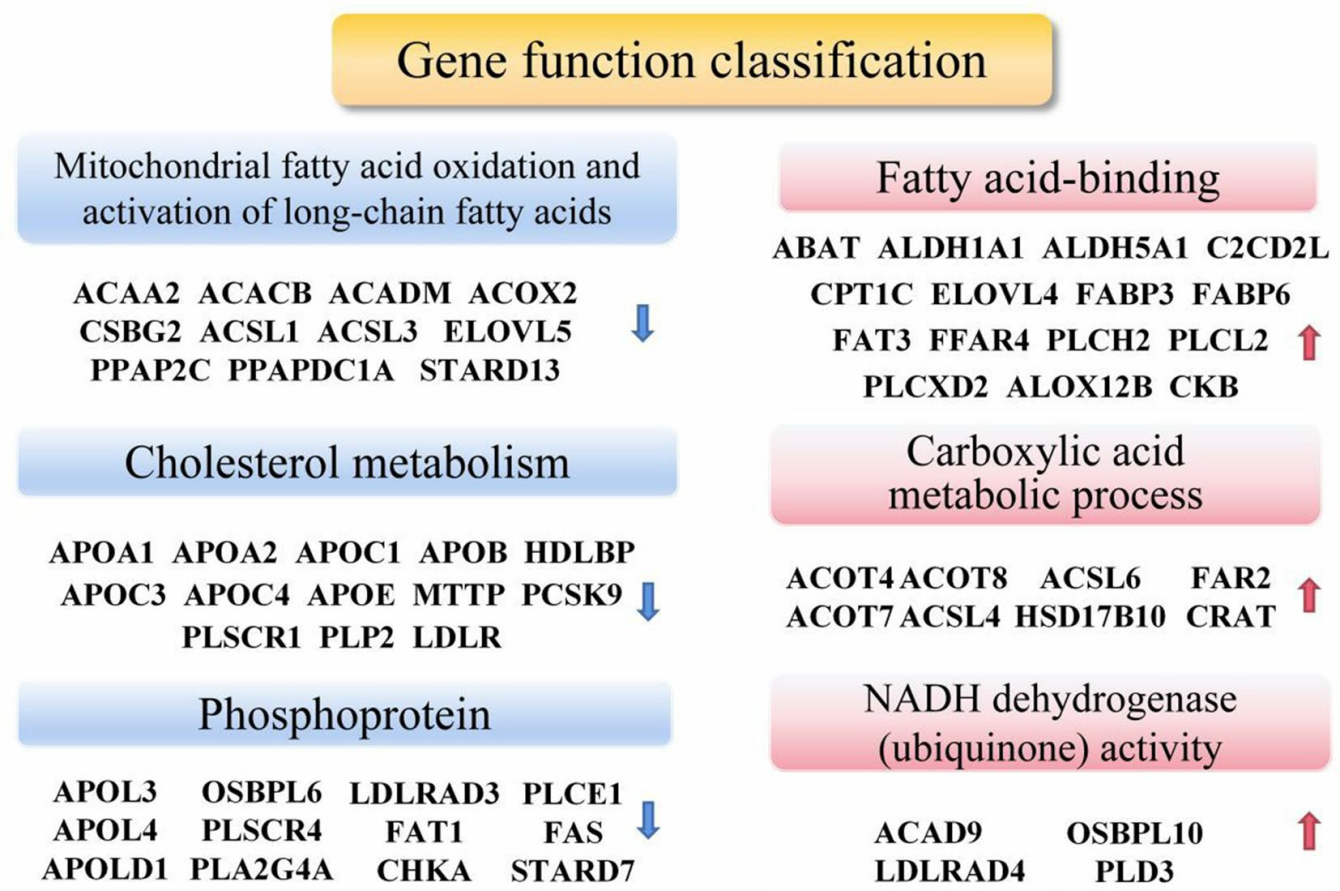
PPI-based classification of 60 differential lipid metabolic proteins

The PPI network consisted of 3 distinct function clusters, each of which contained identical function proteins and was represented by a different color (Fig. 5). The 30 up-regulated proteins were classified to build four visible interaction function clusters (subnetworks). They were related to regulating mitochondrial fatty acid oxidation, and mediating activation of long-chain fatty acids (green), phosphoprotein (red), and cholesterol metabolism (blue), respectively. Among these, apoptosis-involved factors CASP10, CASP7, CASP8, CASP3, and CASP6 contained within red clusters. The network analysis results provided evidence that 30 up-regulated proteins may regulate phosphoprotein, cholesterol metabolism, mitochondrial fatty acid oxidation, and the mediating activation of long-chain fatty acids during early AD pathological processes. Table 7 displays the symbols and full names of the predicted functional anticipated partners in PPI networks. The PPI classification of 60 candidate proteins is shown in Fig. 6.

**Table 7:** The symbols and full names of the predicted proteins in the PPI networks

No.	Accession no.	Protein symbol	Full name	Score
*Up-regulated network*				
1	P80404	ABAT	4-Aminobutyrate aminotransferase, mitochondrial	0.997
2	P30084	ECHS1	Enoyl-CoA hydratase, mitochondrial	0.992
3	Q9BQ95	ECSIT	Evolutionarily conserved signaling intermediate in Toll pathway, mitochondrial	0.994
4	O14842	FFAR1	Free fatty acid receptor 1	0.993
5	P03923	MT-ND6	NADH-ubiquinone oxidoreductase chain 6	0.992
6	Q9Y375	NDUFAF1	Complex I intermediate-associated protein 30, mitochondrial	0.996
7	Q9BU61	NDUFAF3	NADH dehydrogenase [ubiquinone] 1 alpha subcomplex assembly factor 3	0.995
8	O75306	NDUFS2	NADH dehydrogenase [ubiquinone] iron-sulfur protein 2, mitochondrial	0.995
9	Q8IUX1	TMEM126B	Complex I assembly factor TMEM126B, mitochondrial	0.997
10	Q7L0Y3	TRMT10C	tRNA methyltransferase 10 homolog C	0.994
*Down-regulated network*				
1	P11310	ACADM	Medium-chain specific acyl-CoA dehydrogenase, mitochondrial	0.996
2	Q0VD83	APOB	Apolipoprotein B-100	0.996
3	P02649	APOE	Apolipoprotein E	0.996
4	Q92851	CASP10	Caspase-10	0.999
5	O15519	CASP8	Caspase-8	0.999
6	O15519	CFLAR	CASP8 and FADD-like apoptosis regulator	0.999
7	P48023	FADD	FAS-associated death domain protein	0.999
8	P48023	FASLG	Tumor necrosis factor ligand superfamily member 6	0.998
9	Q92851	PCSK9	Proprotein convertase subtilisin/kexin type 9	0.999
10	Q12772	SREBF2	Sterol regulatory element-binding protein 2	0.994

### Correlation analysis of network and core targets screen

**Fig. 7:**
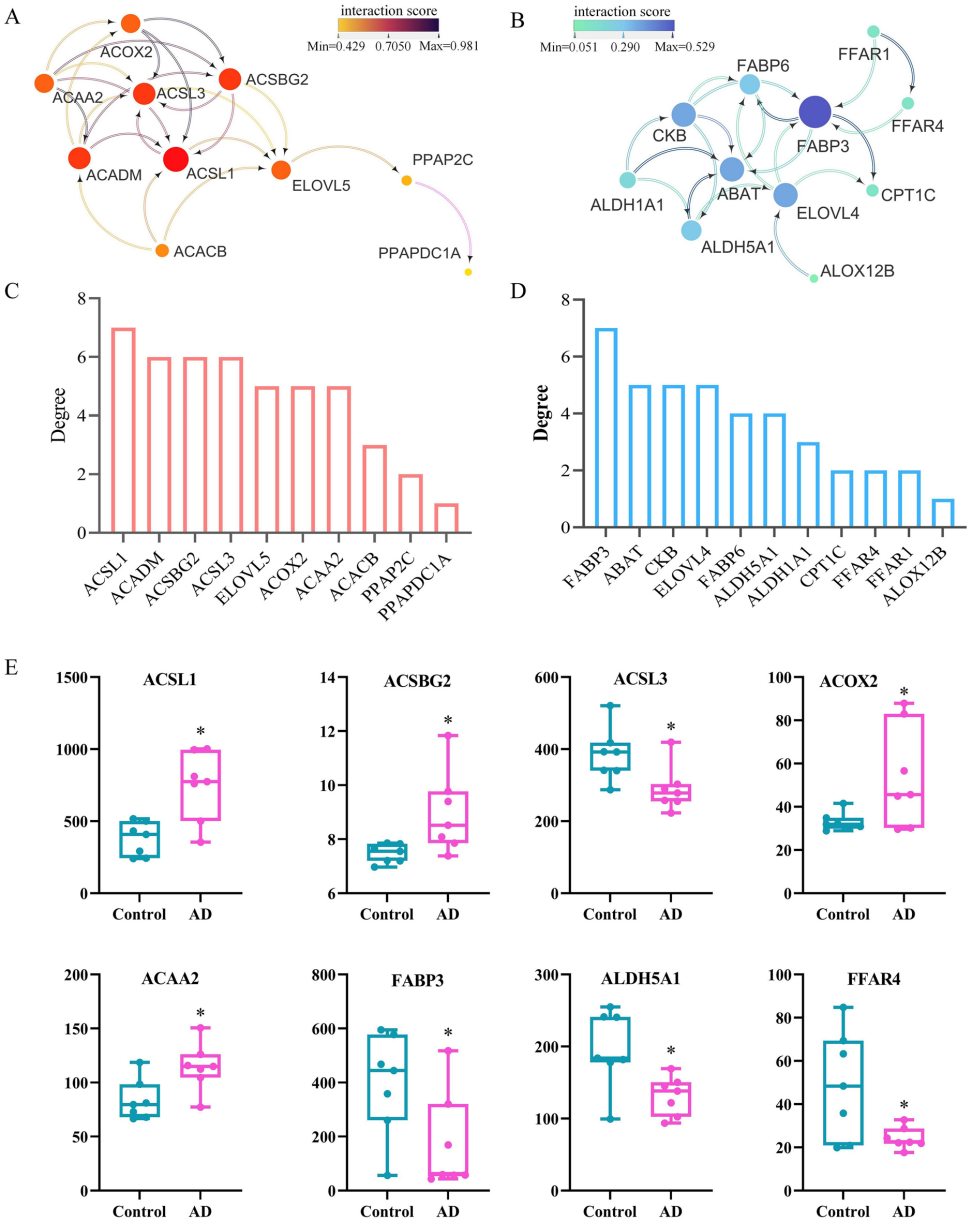
PPI network constructed by protein targets of subnetworks. **a** PPI network of mitochondrial fatty acid oxidation subnetwork. **b** PPI network of the fatty acid-binding subnetwork. Nodes, targets, edges, and interaction among targets. The darker the color and the larger the node, the higher the degree. The color of the edges represents the interaction score. **c** The core targets of the mitochondrial fatty acid oxidation subnetwork were excavated according to the degree. **d** The core targets of the fatty acid-binding subnetwork were excavated according to the degree. **e** Boxplots visualize the expression of the core genes among the control and AD groups

The mitochondrial fatty acid oxidation (Fig. 7a) and lipid transport networks (Fig. 7b) were constructed, respectively. The combined score represents the correlation strength between 2 nodes, which was displayed by the thickness of the edge. The combined score showed the strength of the correlation between the two nodes, as indicated by the thickness of the edge. The darker the color, the stronger the correlation. The average clustering coefficient of mitochondrial fatty acid oxidation and lipid transport network was 0.705 and 0.575, respectively. One of the topological characteristics in this network is degrees of freedom, which reflects the number of connections between distinct nodes, as shown by the size and color of the nodes. As a result, 8 core targets were screened CytoHubba in Cytoscape, including ACSL1, ACSBG2, ACSL3, ACAA2, ACOX2, FABP3, ALDH5A1, FABP6, and FFAR4 (Fig. 7c, d). In addition, Fig. 7e shows relative gene expression of core genes compared to controls. The CTD database and literature reports revealed that all the hub genes targeted AD and AD-related symptoms.

### The relevance of core genes to neurodegenerative diseases

**Fig. 8:**
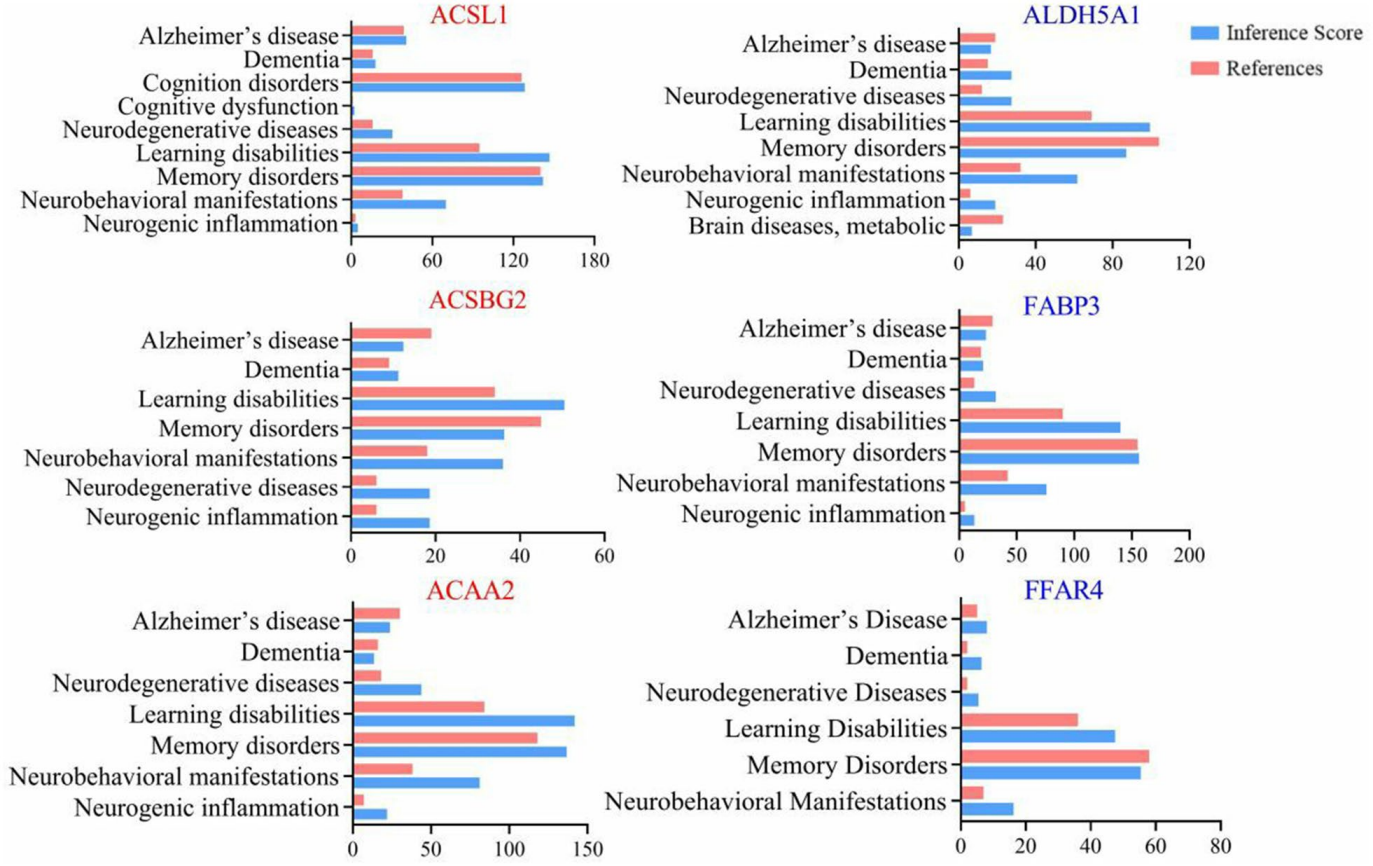
The relevance of core targets to neurodegenerative diseases and early AD-related symptoms

CTD was used to analyze the correlation of the core genes with neurodegenerative diseases and early AD-related symptoms. Inference scores genes for disease relevance based on experimental evidence and co-citation in the literature and summarizes per-gene relevance. Based on experimental data, inference scores revealed the illness significance of each core gene, and references provided the number of publications. These results indicated that all the core genes were associated with early AD neuropathological changes and closely related to memory disorders and learning disabilities, as shown in Fig. 8.

### Drug-targets docking results

**Fig. 9:**
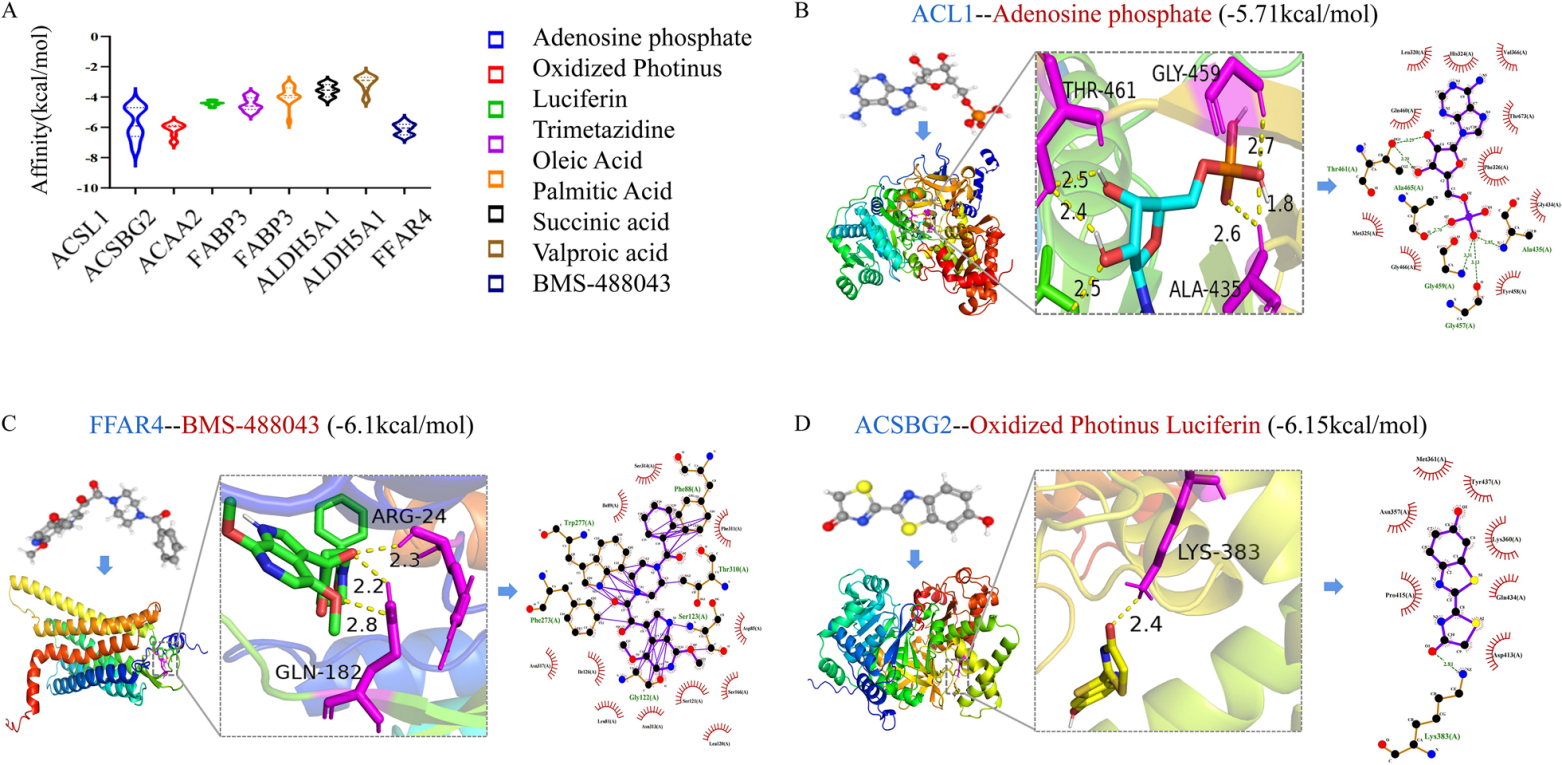
Drug-targets docking results and binding site models. **a** Violin plot of the docking scores; **b** 3D docking conformation of adenosine phosphate with ACSL1. **c** 3D docking conformation of oxidized Photinus luciferin with ACSBG2. **d** 3D docking conformation of BMS-488043 with FFAR4

The potential compounds targeting to 8 core targets were screened, including adenosine phosphate, oxidized Photinus luciferin, trimetazidine, oleic acid, palmitic acid, succinic acid, valproic acid, BMS-488043. Molecular docking was used to further validate the binding capacity of candidate drugs and core targets. The information on candidate compounds, core targets, and their docking results are shown in Table 8. References for drug information were listed. Based on the docking score, we selected the docking results of the compounds that bind best to the target protein for display (Fig. 9). The drug targets docking scores suggested that the binding affinity of oxidized Photinus luciferin to ACSBG2, BMS-488043 to FFAR4, and adenosine phosphate to ACSL1 was higher than others. As a nutraceutical, adenosine phosphate had best binding activity to AD putative core targets, indicating it might also have a potential therapeutic effect in AD.

**Table 8:** The molecular docking results of candidate therapeutic drugs and their directly targeting proteins

Protein	UniProt ID	PDB ID	AlphaFold ID	Drugs	Compound CID	Drug group	Affinity (kcal/mol)	References
ACSL1	P33121		P33121	Adenosine phosphate	6083	Approved, investigational, nutraceutical	− 5.71	[68, 69]
ACSBG2	Q5FVE4		Q5FVE4	Oxidized Photinus luciferin	135398698	Experimental	− 6.15	[70]
ACAA2	P42765	4c2k		Trimetazidine	21109	Experimental	− 4.44	[71]
FABP3	P05413	5hz9		Oleic acid	445639	Approved, Investigational, Vet_approved	− 4.47	[72]
				Palmitic acid	985	Approved	− 3.86	[73]
ALDH5A1	P51649	2w8n		Succinic acid	1110	Approved, Nutraceutical	− 3.54	[74, 75]
				Valproic acid	3121	Approved, Investigational	− 3.08	[76]
FFAR4	Q5NUL3		Q5NUL3	BMS-488043	507806	Investigational	− 6.10	[77]

## Discussion

In recent years, lipid metabolism gradually becomes a major trend in early AD research. GSE39420 datasets were derived from the brain whole-genome RNA expression profile of the posterior cingulate area of 7 sporadic early-onset AD and 7 control subjects [23]. GSE118553 datasets were derived from sporadic entorhinal cortex, frontal cortex, temporal cortex, and cerebellum tissue of 33 AsymAD and 27 control subjects [3]. The tissues of these datasets were derived from the cortex of early AD patients. Thus, they were suitable for the following analysis. The results showed that 30 up-regulated and 30 down-regulated genes of lipid metabolism were all selected from differentially expressed genes from 2 GEO datasets. According to the GO analysis, these proteins were highly enriched in categories such as catalytic activity, cellular process, and metabolic process. Although the pathway analysis showed that some pathways such as inflammation mediated by chemokine and cytokine signaling pathway, 5-Hydroxytryptamine may be involved in the biological process of inducing signs of AD. PPI analysis revealed that several genes/proteins such as ACSL1, ACSBG2, ACAA2, FABP3, ALDH5A1, and FFAR4 might be one of the core genes involved in the biological process.

**Fig. 10:**
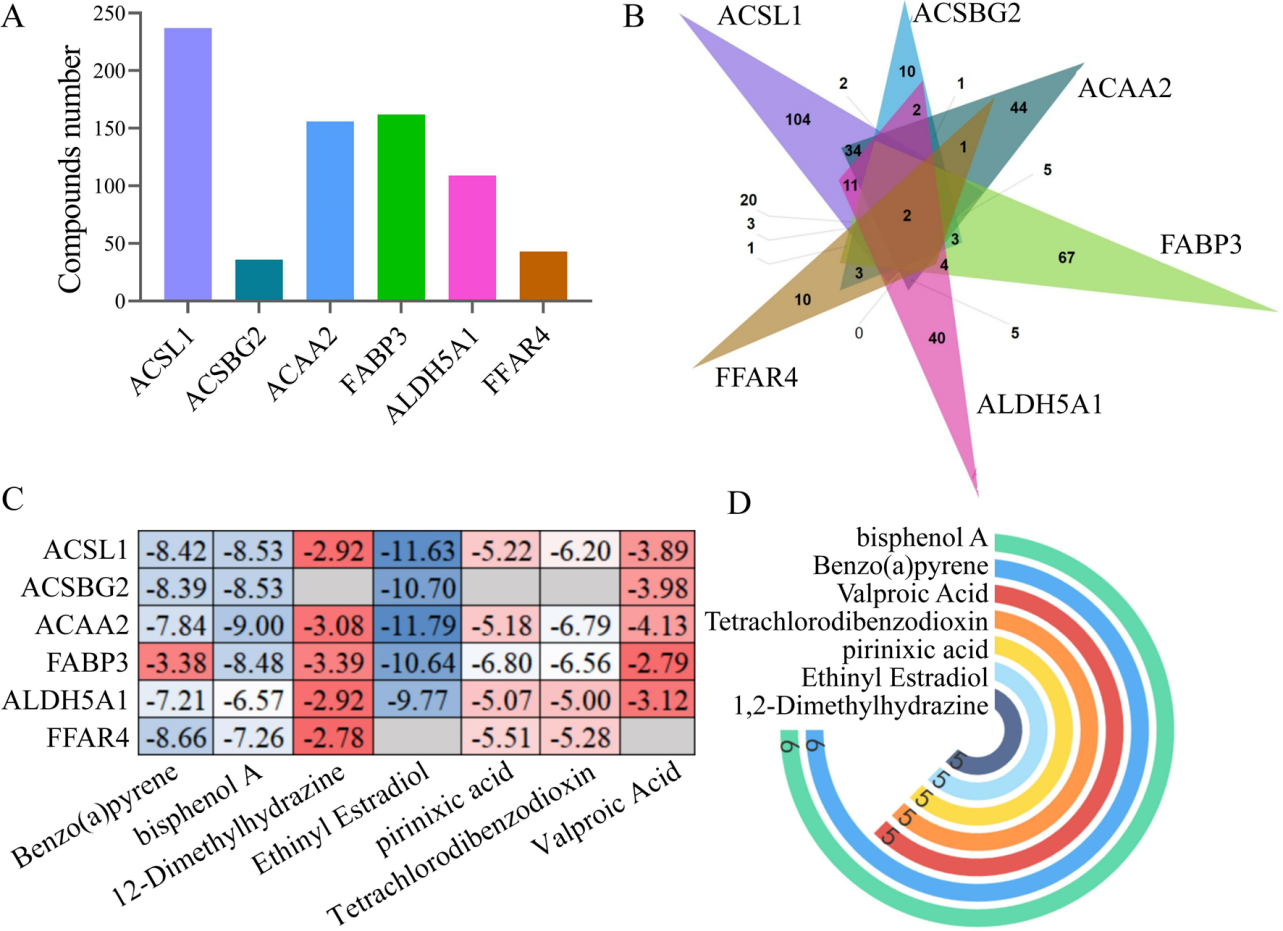
Candidate drugs screen. **a** Venn diagram of candidate compounds to 6 core targets. **b** The total number of candidate compounds to 6 core targets. **c** The heat map was created according to docking scores of candidate drugs and core targets. **d** The number of targets affected by candidate drugs

The literature review was performed by the CTD database to investigate potential compounds for early AD therapy. According to the literature, the drugs that can regulate core gene expression were screened, including bisphenol A, benzo(a)pyrene, valproic acid, tetrachlorodibenzodioxin, pirinixic acid, ethinyl estradiol, and 1,2-Dimethylhydra zine. The regulation of candidate compounds to corresponding core targets is listed in Table 9. The intersection of compounds corresponding to 6 core targets is shown in Fig. 10a. The statistics of the total number of potential compounds to core targets are shown in Fig. 10b. Molecular docking study of 7 overlap compounds and 6 core targets was performed, and the docking scores were presented by a heat map, as shown in Fig. 10c. The intersection of 6 core targets and their corresponding compounds was taken (Fig. 10d). It is widely assumed that the lower the energy when the ligand’s conformation binding to the receptor remains stable, the higher the potential of activation. The results indicated that the active compounds, especially ethinyl estradiol and bisphenol A, had good binding activity to the core targets, indicating the probable role of candidate compounds for early AD treatment.

**Table 9:** The regulation of candidate drugs to core targets

No.	Chemical name	PubChem ID	ACSL1	ACSBG2	ACAA2	FABP3	ALDH5A1	FFAR4
1	Benzo(a)pyrene	2336	Increases expression	Affects methylation	Affects expression, affects reaction	Affects methylation	Affects methylation and expression	Decreases expression, increases methylation
2	Bisphenol A	6623	Increases expression	Decreases expression, affects methylation	Decreases expression	Affects expression	Increases methylation, affects expression	Decreases expression, increases methylation
3	1,2-Dimethylhydrazine	1322	Decreases expression		Decreases expression	Increases expression	Decreases expression	Decreases expression
4	Ethinyl estradiol	5991	Decreases expression	Affects expression	Decreases expression	Affects expression	Increases expression	
5	Pirinixic acid	5694	Increases expression		Increases expression	Increases expression	Increases expression	Increases expression
6	Tetrachlorodibenzodioxin	15625	Decreases expression		Affects expression	Increases expression	Decreases expression	Decreases expression
7	Valproic acid	3121	Increases expression	Affects expression, increases methylation	Decreases methylation, increases expression	Increases expression	Affects expression

**Fig. 11:**
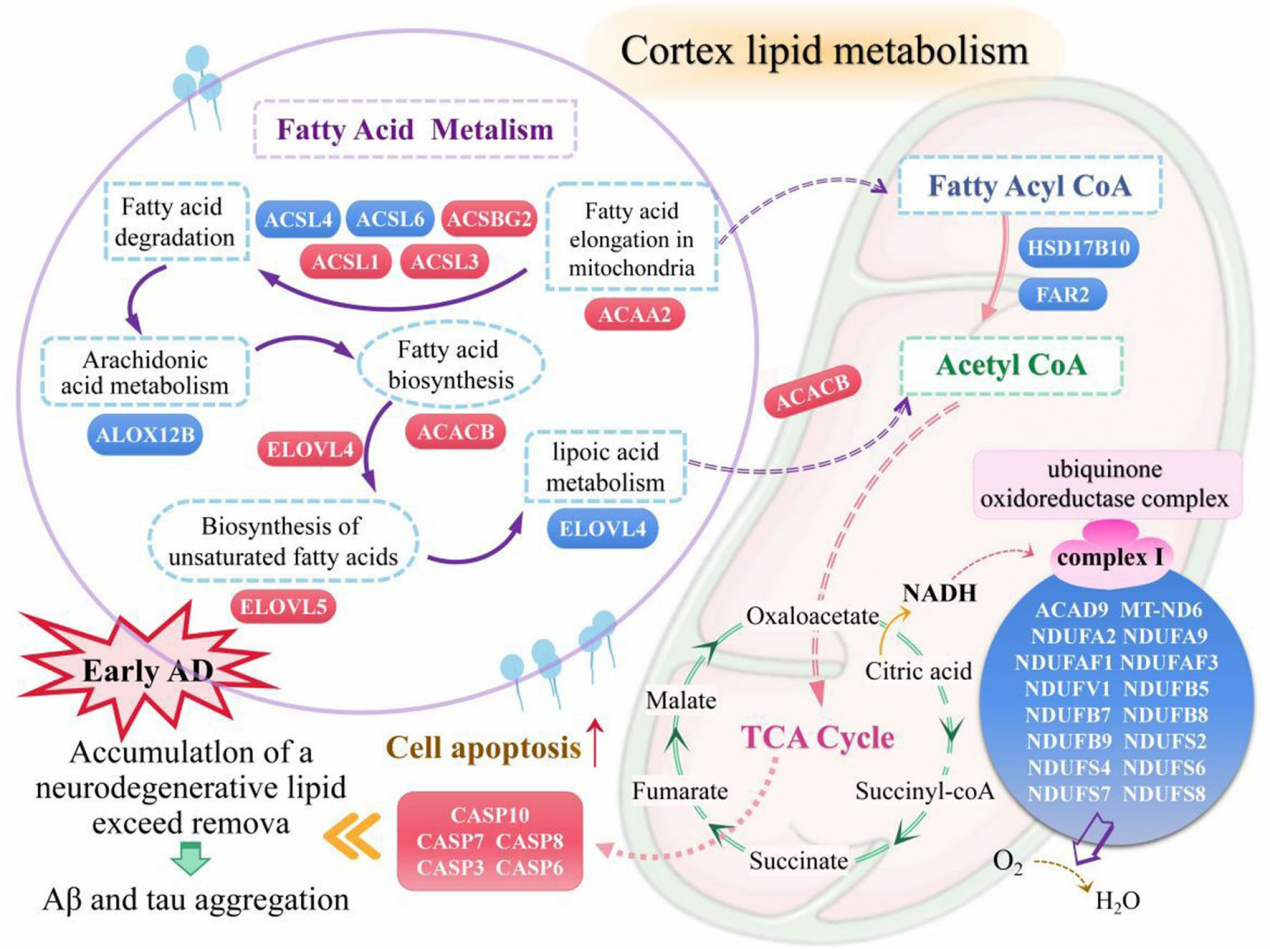
A putative schematic model of the regulation mechanism of the lipid metabolic genes on the cerebral cortex in early AD patients

Based on the molecular functions of these lipid metabolic genes on the cerebral cortex in early AD patients, we made a taxonomic profile and a putative schematic model of the regulation mechanism of the lipid metabolic genes on the cerebral cortex in early AD patients (Fig. 11).

In the cerebral cortex of AD rats, sphingolipid metabolism, glycerophospholipid metabolism, linoleic acid metabolism, and alpha-linolenic acid metabolism were discovered to be dysregulated. Lipidomics analysis of PLB4 hBACE1 knock-in mouse model of AD showed decreases in the phosphatidylethanolamine content of the cortex [24]. The lipid transporter Spns2 could promote pro-inflammatory polarization of microglia through the NFκB pathway and play a crucial role in AD pathogenesis [25]. Cholesterol promoted Aβ_42_ aggregation by enhancing its primary nucleation rate through a heterogeneous nucleation pathway [26]. Aβ_42_ aggregation could be promoted by cholesterol via a heterogeneous nucleation pathway.

Aβ peptides can also interact with cholesterol and gangliosides, which potentiate the formation of fibrils and Aβ oligomers [12]. The cytotoxic of Aβ was principally mediated by modulation of the activity of phospholipases such as PLA2, PLD2, and PLC [13]. The accumulation of amyloid-beta peptides (Aβs), the formation of neurofibrillary tangles containing the tau protein, and the degeneration of neurons have all been identified as key processes underlying AD [27]. Ganglioside GM1 has been demonstrated to cause particular alterations in the spatial structure of Aβ, resulting in increased peptide accumulation and a negative impact [7]. Lipid rafts were seen as primary targets for Aβ peptide cytotoxicity. Lipids interfere with amyloid clearance by affecting proteasome and autophagy activities [6]. Lipid rafts disrupted the aberrant degradative autophagic-lysosomal pathway of Aβ, and were implicated in the production of Aβ oligomers and Aβ peptides [28]. The accumulation of amyloid deposits of Aβ peptides in the AD brain is related to abnormal lipid metabolism in the cortex.

In network analysis, we found a subnetwork was related to lipid transport. The network contains many lipid transport-related proteins including ApoB, APOA1, APOA1, and LDLR. Previous studies have demonstrated significant alterations of lipid transport in early AD patients’ cortex. Lipid transport is one of the important factors for lipid metabolism [29, 30]. ApoB plays a critical role in pre-clinical AD. Lower ApoB level was observed in the cerebrospinal fluid of AD patients. The lower level of serum ApoB was associated with decreased Aβ_42_ and Aβ_42/40_ and increased p-tau/Aβ_42,_ t-tau/Aβ_42_, and p-tau [31].

The deletion of the APOA1 gene can reduce the concentration of insoluble Aβ_40_ and Aβ_42_ [32]. Button EB et al. found that ApoA1 deficiency increases Aβ deposition, astrogliosis, and amyloid-associated astrocyte reactivity in the cortex and hippocampus of APP/PS1 mice [33]. Compared to wild-type control, ApoA1 level significantly increased to 187% of 5xFAD mice. Increased levels of ApoA1 result in increased HDL cholesterol [32]. In the Tg2576 model, loss of apoA-I was linked with enhanced Aβ elimination and decreased Aβ pathology [34].

LDLR has the highest affinity for ApoE and plays an important role in brain cholesterol metabolism [35]. LDLR is one of the molecules that is involved in the clearance of amyloid proteins in the AD brain [36]. LDLR-deficient mice developed age-dependent cerebral β-amyloidosis. Cao et al. found an increase in Aβ deposition and apoE expression in LDLR-deficient Tg2576 mice. LDLR-deficient Tg2576 mice showed more spatial learning deficits than LDLR-intact Tg2576 mice [37]. Abisambra et al. found that LDLR and APP protein levels were directly proportional in PSAPP mice compared to controls. LDLR protein increased fourfold in H4-APP cells compared to H4 controls. The results suggested that APP overexpression can abrogate LDLR transport to the cell membrane, reducing the transport of LDLR to the plasma membrane, and leading to up-regulation of LDLR transcription and protein levels.

PLD3 (phospholipase D family, member 3) gene encoded a lysosomal protein that could catalyze membrane phospholipids’ hydrolysis 14. The exon11 of PLD3 was associated with AD risk. PLD3 mRNA levels in the human prefrontal cortex were shown to be inversely associated with the degree of amyloid pathology and the rate of cognitive impairment in 531 patients [38]. PLD3 mRNA expression levels were inversely related to hippocampal β-amyloid load, which decreased PLD3 and affected amyloid-β levels in AD cell models via the autophagy-dependent mTOR signaling pathway [39]. The lack of function at PLD3 protein could increase APP processing [40]. PLD3, which may be co-immunoprecipitated with APP in cultured cells, has been shown to operate as a negative regulator of APP processing in previous investigations [41]. The PLD3 gene was highly expressed in healthy brains, particularly in certain brain areas that were sensitive to AD pathology, such as the frontal, temporal, and occipital cortices, as well as the hippocampus, but it is markedly decreased in neurons in AD brains [42]. In this study, the expression level of PLD3 was decreased in AD cerebral cortex. These findings suggested that PLD3 may have a protective effect on AD pathogenesis by participating in APP trafficking. Furthermore, decreased PLD3 could impair the endosomal-lysosomal system, which has been hypothesized as an alternate mechanism of AD. On concluding, PLD3 is an AD risk gene linked to the neurobiology of lysosomal dysfunction and β-amyloid pathology in AD.

The LDL receptor (LDLR) family members, including LDLR and LDLR-related proteins, are cell surface receptors with comparable structural and functional properties. Evidence from cellular and animal research suggested that members of the LDLR family could contribute to AD pathogenesis by regulating Aβ buildup or neurodegenerative processes. Some can recognize ApoE and Aβs for cellular trafficking and signaling pathways [42]. LRP1 may aid Aβ absorption into cells by collaborating with other A-binding proteins, heparan sulfate proteoglycan, for example [43]. Several experiments have shown that LRP1 influenced A metabolism and brain homeostasis via both apoE isoform-dependent and -independent pathways. Using flow cytometry, Bilousova et al. measured the ApoE receptors LDLR in synaptosomes, indicating up-regulation of LDLR in early and late-stage AD [44]. Previous research has revealed that LDLR transports Aβ into synapses, contributing to synaptic Aβ accumulation [45]. LDLR was expressed in various cells, including neurons, astrocytes, and vasculatures, and its levels may change depending on the cell type in AD [46]. Some studies have found that LRLR mRNA levels were higher in AD patients’ temporal cortex and hippocampus or in demented people [47]. In this study, the expression level of LDLR was elevated in the early AD cerebral cortex. On concluding, LDLR may play a role in increasing Aβ accumulation and toxicity in AD pathogenesis.

PLA2G4A (phospholipase A2, group IVA [cytosolic, calcium-dependent]) is one of three main phospholipases found in the brain [48]. Aβ oligomers bind to cellular prion proteins (PrPC) in synaptosomes, leading to increased cholesterol concentrations, PLA2G4A activation, and translocation to lipid rafts [49]. Esterification of cholesterol was a key factor in the dispersal of Aβ-induced signaling platforms involved in the activation of PLA2G4A and synapse degeneration, which aggravates the formation of impaired cognition [50]. The esterification of cholesterol is the cause of the dissemination of Aβ-induced signaling platforms, implicated in the activation of PLA2G4A and synapse degradation, which exacerbates the genesis and progression of AD [51]. In numerous neurodegenerative disorders, both PLA2G4A activity and expression levels were increased [52, 53]. In this study, the expression level of PLA2G4A was also increased in the early AD cortex. On concluding, PLA2G4A impacts the development of AD by affecting the esterification of cholesterol.

Belonging to the fatty acid-binding protein (FABP) family, FABP3 in the brain may regulate the neuronal membrane’s lipid composition. FABP3 may influence synaptic plasticity and cholinergic activity, glutamatergic and GABAergic inhibitory interneurons, and notably intracellular lipid transport [54]. FABP3 may also affect dopamine D2R function in the striatum and anterior cingulate cortex (ACC), which is critical for cognitive coordination [55]. FABP3 may regulate Aβ and alpha-synuclein (Syn) aggregation through ARA, resulting in the development of Aβ plaques [56]. FABP3 was expressed in the cerebral neocortex and hippocampal CA1 and CA2 region, especially in dopaminergic, acetyl cholinergic, and glutamatergic neurons. Overexpression of FABP3 was linked to tau pathology and neurodegeneration [57]. There is a link between high FABP3 levels and atrophy of critical brain regions in patients with abnormal amyloid concentrations [58]. According to the latest research, FABP3 concentrations in CSF fluid were shown to be significantly greater in AD patients [59]. In this study, the expression level of FABP3 was decreased in the AD cortex. This opposite result may be due to various tissues and their different metabolic requirements. On concluding, FABP3 inhibits GABAergic transmission and causes neuronal dysfunction by acting on phosphorylated Tau protein [60].

ACSL1 was an ATP-dependent AMP-binding enzyme that catalyzed the conversion of long-chain fatty acids to their active form acyl-CoAs for cellular lipid synthesis and breakdown via beta-oxidation. At the intersection of anabolic and catabolic pathways, fatty acids were involved in the formation of phospholipids and triacylglycerols and were capable of β-oxidation [61]. Growing evidence suggested that fatty acids were involved in pathological states such as neurodegenerative illnesses, mental disorders, stroke, and trauma. Fatty acids increase neuronal activity, synaptogenesis, and neurogenesis while also preventing neuroinflammation and apoptosis [62]. In this study, ACSL1 expression was elevated in the cerebral cortex, which may benefit brain growth and improve cognitive function. On concluding, ACSL1 could promote neuronal activity synaptogenesis and neurogenesis while avoiding AD-induced neuroinflammatory damage and death.

Produced by HSD17B10 gene, hydroxysteroid (17β) dehydrogenase 10 (HSD10) is a mitochondrial NAD^+^-dependent dehydrogenase, playing a key role in the degradation of isoleucine and the metabolism of neuroactive substances [63]. This essential enzyme’s diversity in catalytic activity and ability to bind other proteins and peptides are two remarkable properties. HSD17B10 is essential in mitochondrial fatty acid metabolism because it serves as an active site for various substrates, including steroids, fatty acids, bile acids, and xenobiotics [64]. Although this enzyme was present in almost all tissues, its expression levels varied among brain regions [65]. In the hippocampi of AD patients and mice models, HSD10 levels were elevated. However, in this study, the expression level of HSD17B10 was decreased in the cerebral cortex. HSD17B10 was involved in AD pathogenesis as the endoplasmic reticulum-associated Aβ-binding protein (ERAB) and as Aβ-binding alcohol dehydrogenase (ABAD). Although HSD17B10 was also important in mitochondrial tRNA maturation, according to the findings [66], the main contribution of HSD17B10 to AD may be its overexpression in AD patients which disrupts the homeostasis of neurosteroid metabolism [67].

Although various analysis methods were verified mutually, we do acknowledge the limitations of this work. This study solely relies on existing gene expression data from public databases and lacks experimental validation. Further in vitro and in vivo studies are needed to confirm the role of key proteins and active compounds in early AD. The importance and relative abundance of key targets need to be validated in more human samples and by more methods. Although this study relies on Microarrays for gene expression analysis, the use of predefined probe sets may limit the identification of unknown genes. We plan to integrate RNA sequencing in future investigations, enhancing the comprehensive profiling of lipid metabolism-related genes in early AD and overcoming Microarrays limitations for a more thorough exploration of the molecular landscape in early AD.

## Conclusion

In conclusion, 60 differently expressed genes related to cortical lipid metabolism were screened. These early AD risk proteins could modulate brain lipid homeostasis by the PPAR signaling pathway, glycerophospholipid metabolism, adipocytokine signaling pathway, fatty acid biosynthesis, fatty acid degradation, ferroptosis, biosynthesis of unsaturated fatty acids, and fatty acid elongation. 6 core genes targets for early AD diagnosis were screened, which had a high binding affinity with compounds including adenosine phosphate, oxidized Photinus luciferin, BMS-488043, and drugs, especially bisphenol A, benzo(a)pyrene, and ethinyl estradiol. These compounds and candidate drugs may have potential therapeutic effects on cortical lipid metabolism disorder of early AD.

### Supplementary Information

**Additional file 1: Table S1.** The molecular function and biological process category of 30 down-regulated genes. **Table S2.** The molecular function and biological process category of 30 up-regulated genes.

## Data Availability

The raw data of our study were downloaded from GEO dataset (https://portal.gdc.cancer.gov/).
