# Supplementary material for: Cortical lipid metabolic pathway alteration of early Alzheimer’s disease and candidate drugs screen
Source: Eur J Med Res. 2024 Mar 25;29:199. doi: 10.1186/s40001-024-01730-w (PMC10962147; doi:10.1186/s40001-024-01730-w)
Supplement: Supplementary file 1 — Additional file 1: Table S1. The molecular function and biological process category of 30 down-regulated genes. Table S2. The molecular function and biological process category of 30 up-regulated genes. [file 40001_2024_1730_MOESM1_ESM.docx]

# Additional Tables

**Table S1** The molecular function and biological process category of 30 downregulated genes in cerebral cortex of AD patients.

| NO | Symbol | Gene name | molecular function | biological process |
| --- | --- | --- | --- | --- |
| 1 | ACAD9 | acyl-CoA dehydrogenase family member 9 | acyl-CoA dehydrogenase activity; carbohydrate derivative binding; purine nucleotide binding; amide binding | long-chain fatty acid metabolic process; medium-chain fatty acid metabolic process; mitochondrial respiratory chain complex I assembly |
| 2 | ACOT4 | acyl-CoA thioesterase 4 | acyl-CoA hydrolase activity; carboxylic ester hydrolase activity; myristoyl-CoA hydrolase activity; palmitoyl-CoA hydrolase activity; succinyl-CoA hydrolase activity | acyl-CoA metabolic process; fatty acid biosynthetic process; fatty acid metabolic process; long-chain fatty acid metabolic process; short-chain fatty acid metabolic process; very long-chain fatty acid metabolic process |
| 3 | ACOT7 | acyl-CoA thioesterase 7 | carbohydrate derivative binding;purine nucleotide binding;thiolester hydrolase activity;anion binding;amide binding | acyl-CoA metabolic process;long-chain fatty acid metabolic process;fatty acid biosynthetic process |
| 4 | ACOT8 | Acyl-coenzyme A thioesterase 8 | acetoacetyl-CoA hydrolase activity; acetyl-CoA hydrolase activity; acyl-CoA hydrolase activity; carboxylic ester hydrolase activity; choloyl-CoA hydrolase activity; CoA hydrolase activity | acyl-CoA metabolic process; bile acid biosynthetic process; dicarboxylic acid catabolic process; fatty acid beta-oxidation using acyl-CoA oxidase; fatty acid catabolic process; protein targeting to peroxisome |
| 5 | ACSL4 | Long-chain-fatty-acid--CoA ligase 4 | acyl-CoA ligase activity; arachidonate-CoA ligase activity; long-chain fatty acid-CoA ligase activity; very long-chain fatty acid-CoA ligase activity | acyl-CoA metabolic process;long-chain fatty acid metabolic process;neuron differentiation;fatty acid derivative metabolic process |
| 6 | ACSL6 | acyl-CoA synthetase long-chain family member 6 | acyl-CoA ligase activity; arachidonate-CoA ligase activity; enzyme binding; long-chain fatty acid-CoA ligase activity | acyl-CoA metabolic process; long-chain fatty acid metabolic process;lipid biosynthetic process;very long-chain fatty acid metabolic process;fatty acid derivative metabolic process |
| 7 | ALDH1A1 | aldehyde dehydrogenase 1 family member A1 | aldehyde dehydrogenase (NAD+) activity; benzaldehyde dehydrogenase (NAD+) activity; GTPase activator activity; NAD binding | cellular aldehyde metabolic process; ethanol oxidation; fructose catabolic process to hydroxyacetone phosphate and glyceraldehyde-3-phosphate; oxidation-reduction process |
| 8 | ALDH5A1 | aldehyde dehydrogenase 5 family member A1 | succinate-semialdehyde dehydrogenase (NAD+) activity; succinate-semialdehyde dehydrogenase [NAD(P)+] activity | central nervous system development; gamma-aminobutyric acid catabolic process; glutamate metabolic process; neurotransmitter catabolic process |
| 9 | ALOX12B | arachidonate 12-lipoxygenase, 12R type | oxidoreductase activity, acting on single donors with incorporation of molecular oxygen, incorporation of two atoms of oxygen | long-chain fatty acid metabolic process;lipid oxidation;unsaturated fatty acid metabolic process |
| 10 | C2CD2L | Phospholipid transfer protein C2CD2L | insulin binding; phosphatidylinositol binding; phosphatidylinositol transfer activity | [positive regulation of insulin secretion involved in cellular response to glucose stimulus](https://www.ebi.ac.uk/QuickGO/term/GO:0035774) |
| 11 | CKB | Creatine kinase B-type | kinase activity | cellular modified amino acid metabolic process; organophosphate biosynthetic process |
| 12 | CPT1C | carnitine palmitoyltransferase 1C | [carnitine O-palmitoyltransferase activity](https://www.ebi.ac.uk/QuickGO/term/GO:0004095) | fatty acid beta-oxidation; fatty acid metabolic process; regulation of postsynaptic membrane neurotransmitter receptor levels |
| 13 | CRAT | Carnitine O-acetyltransferase | O-acyltransferase activity;palmitoyltransferase activity | cellular modified amino acid metabolic process; fatty acid metabolic process |
| 14 | ELOVL4 | Elongation of very long chain fatty acids protein 4 | acetyltransferase activity;O-acyltransferase activity | cellular modified amino acid metabolic process;cellular nitrogen compound metabolic process |
| 15 | FABP3 | fatty acid binding protein 3 | transferase activity, transferring acyl groups other than amino-acyl groups | fatty acid biosynthetic process;very long-chain fatty acid metabolic process;sphingolipid biosynthetic process |
| 16 | FABP6 | fatty acid binding protein 6 | [lipid binding](https://www.ebi.ac.uk/QuickGO/term/GO:0008289) | lipid metabolic process; lipid transport; triglyceride catabolic process |
| 17 | FADS6 | fatty acid desaturase 6 | [oxidoreductase activity](https://www.ebi.ac.uk/QuickGO/term/GO:0016491) | [fatty acid biosynthetic process](https://www.ebi.ac.uk/QuickGO/term/GO:0006633) |
| 18 | FAR2 | fatty acyl-CoA reductase 2 | [NADH dehydrogenase (ubiquinone) activity](https://www.ebi.ac.uk/QuickGO/term/GO:0008137); alcohol-forming fatty acyl-CoA reductase activity;  fatty-acyl-CoA reductase (alcohol-forming) activity | lipid metabolic process; long-chain fatty-acyl-CoA metabolic process |
| 19 | FAT3 | FAT atypical cadherin 3 | oxidoreductase activity | acyl-CoA metabolic process;fatty acid derivative metabolic process |
| 20 | FFAR4 | free fatty acid receptor 4 | arrestin family protein binding; fatty acid binding; G protein-coupled receptor activity | brown fat cell differentiation; G protein-coupled receptor signaling pathway; negative regulation of interleukin-1 beta production; positive regulation of brown fat cell differentiation; positive regulation of cAMP- mediated signaling; white fat cell differentiation |
| 21 | HSD17B10 | 3-hydroxyacyl-CoA dehydrogenase type-2 | 17-beta-hydroxysteroid dehydrogenase (NAD+) activity; 3-hydroxy-2- methylbutyryl-CoA dehydrogenase activity; 3-hydroxyacyl-CoA dehydrogenase activity | bile acid biosynthetic process; fatty acid beta-oxidation; lipid metabolic process; mitochondrial tRNA methylation; mitochondrial tRNA processing |
| 22 | LDLRAD4 | low density lipoprotein receptor class A domain containing 4 | [R-SMAD binding](https://www.ebi.ac.uk/QuickGO/term/GO:0070412) | negative regulation of pathway-restricted SMAD protein phosphorylation; negative regulation of transforming growth factor beta receptor signaling pathway |
| 23 | OSBPL10 | oxysterol binding protein like 10 | SMAD binding | negative regulation of protein phosphorylation; transforming growth factor beta receptor signaling pathway; protein phosphorylation |
| 24 | PLCH2 | phospholipase C eta 2 | cholesterol binding;lipid transporter activity; phosphatidylinositol phospholipase C activity | inositol phosphate metabolic process; lipid catabolic process; phosphatidylinositol-mediated signaling |
| 25 | PLCL2 | phospholipase C like 2 | GABA receptor binding; inositol 1,4,5 trisphosphate binding; phosphatidylinositol phospholipase C activity | lipid metabolic process; negative regulation of cold-induced thermogenesis; phosphatidylinositol-mediated signaling; regulation of synaptic transmission, GABAergic |
| 26 | PLCXD2 | phosphatidylinositol specific phospholipase C X domain containing 2 | phospholipase C activity; phosphoric diester hydrolase activity | phosphatidylinositol-mediated signaling |
| 27 | PLD3 | phospholipase D family member 3 | phospholipase D activity; single-stranded DNA 5'-3' exodeoxyribonuclease activity | lipid catabolic process; signal transduction |
| 28 | PLPPR2 | phospholipid phosphatase related 2 | lipid phosphatase activity; phosphatase activity; phosphatidate phosphatase activity | phospholipid dephosphorylation; phospholipid metabolic process; signal transduction |
| 29 | PLPPR5 | phospholipid phosphatase related 5 | lipid phosphatase activity; phosphatase activity; phosphatidate phosphatase activity | phospholipid dephosphorylation; phospholipid metabolic process; positive regulation of neuron projection development |

**Table S2** The molecular function and biological process category of 30 upregulated genes in cerebral cortex of AD patients.

| NO | Symbol | Gene name | molecular function | biological process |
| --- | --- | --- | --- | --- |
| 1 | ACAA2 | acetyl-CoA acyltransferase 2 | fatty acid beta-oxidation | acetyltransferase activity |
| 2 | ACACB | acetyl-CoA carboxylase beta | acetyl-CoA carboxylase activity; ATP binding; biotin binding; identical protein binding; metal ion binding | acetyl-CoA metabolic process; fatty acid biosynthetic process; negative regulation of fatty acid beta-oxidation; positive regulation of cellular metabolic process; positive regulation of lipid storage; regulation of cholesterol biosynthetic process |
| 3 | ACOX2 | acyl-CoA oxidase 2 | fatty acid beta-oxidation;lipid homeostasis;very long-chain fatty acid metabolic process | carboxylic acid binding; oxidoreductase activity, acting on the CH-CH group of donors; lipid binding |
| 4 | ACSBG2 | acyl-CoA synthetase bubblegum family member 2 | acyl-CoA hydrolase and ligase activity; arachidonate-CoA ligase activity; CoA-ligase activity; decanoate-CoA ligase activity; long-chain fatty acid-CoA ligase activity | fatty acid metabolic process; long-chain fatty acid biosynthetic process; long-chain fatty-acyl-CoA biosynthetic process |
| 5 | ACSL1 | Long-chain-fatty-acid--CoA ligase 1 | acyl-CoA metabolic process;long-chain fatty acid metabolic process;lipid biosynthetic process;very long-chain fatty acid metabolic process;fatty acid derivative metabolic process | ligase activity, forming carbon-sulfur bonds |
| 6 | ACSL3 | Long-chain-fatty-acid--CoA ligase 3 | acyl-CoA metabolic process;long-chain fatty acid metabolic process;neuron differentiation;fatty acid derivative metabolic process | ligase activity, forming carbon-sulfur bonds |
| 7 | APOC1 | apolipoprotein C1 | triglyceride metabolic process; lipid catabolic process; regulation of lipase activity; cholesterol transport; regulation of lipid metabolic process; protein-containing complex disassembly | carboxylic acid binding;enzyme inhibitor activity; lipid binding;phospholipase activity |
| 8 | APOC4 | apolipoprotein C4 | lipid storage; regulation of localization; triglyceride homeostasis; positive regulation of biological process | lipid metabolic process; positive regulation of sequestering of triglyceride; triglyceride homeostasis; very-low-density lipoprotein particle assembly; very-low-density lipoprotein particle clearance |
| 9 | APOL3 | apolipoprotein L3 | lipid binding; lipid transporter activity | inflammatory response; lipoprotein metabolic process; positive regulation of I-kappaB kinase/NF-kappaB signaling |
| 10 | APOL4 | apolipoprotein L4 | [lipid binding](https://www.ebi.ac.uk/QuickGO/term/GO:0008289) | lipid metabolic process; lipid transport; lipoprotein metabolic process |
| 11 | APOLD1 | apolipoprotein L domain containing 1 | [lipid binding](https://www.ebi.ac.uk/QuickGO/term/GO:0008289) | lipid transport; lipoprotein metabolic process; regulation of endothelial cell differentiation |
| 12 | CHKA | Choline kinase alpha | fatty acid biosynthetic process;very long-chain fatty acid metabolic process;sphingolipid biosynthetic process | transferase activity, transferring acyl groups other than amino-acyl groups |
| 13 | ELOVL5 | ELOVL fatty acid elongase 5 | extrinsic apoptotic signaling pathway in absence of ligand; neuron apoptotic process; positive regulation of apoptotic signaling pathway; stress-activated MAPK cascade | transmembrane signaling receptor activity;tumor necrosis factor binding |
| 14 | FAS | Fatty acid synthase | calmodulin binding; identical protein binding; kinase binding; signaling receptor activity | Fas signaling pathway; immune response; motor neuron apoptotic process; necroptotic signaling pathway; regulation of stress-activated MAPK cascade; signal transduction |
| 15 | FAT1 | FAT atypical cadherin 1 | cell-cell adhesion; calcium ion binding | cell adhesion; cell-cell adhesion; cell-cell signaling; cell migration; homophilic cell adhesion via plasma membrane adhesion molecules |
| 16 | HDLBP | high density lipoprotein binding protein | cadherin binding; lipid binding; mRNA binding; RNA binding | cholesterol metabolic process; high-density lipoprotein particle clearance; lipid transport |
| 17 | LDLR | low density lipoprotein receptor | amyloid-beta binding; low-density lipoprotein particle binding; low-density lipoprotein particle receptor activity; neuropeptide binding; transmembrane signaling receptor activity | negative regulation of amyloid-beta formation; negative regulation of neurofibrillary tangle assembly; negative regulation of neuron death; negative regulation of tau-protein kinase activity; negative regulation of triglyceride catabolic process; neuropeptide signaling pathway |
| 18 | LDLRAD3 | low density lipoprotein receptor class A domain containing 3 | [amyloid-beta binding](https://www.ebi.ac.uk/QuickGO/term/GO:0001540) | receptor-mediated endocytosis; regulation of protein processing |
| 19 | OSBPL6 | oxysterol binding protein like 6 | cholesterol binding; lipid binding; sterol binding; sterol transporter activity | cholesterol binding;lipid transporter activity; bile acid biosynthetic process; regulation of cholesterol transport |
| 20 | PLA2G4A | phospholipase A2 group IVA | glycerophospholipid catabolic process | phospholipase A2 activity;calcium ion binding;calcium-dependent phospholipid binding |
| 21 | PLCE1 | phospholipase C epsilon 1 | guanyl-nucleotide exchange factor activity; metal ion binding; phosphatidylinositol phospholipase C activity; phospholipase C activity | activation of MAPK activity; lipid catabolic process; phospholipase C-activating G protein-coupled receptor signaling pathway; Ras protein signal transduction; regulation of G protein-coupled receptor signaling pathway; regulation of protein kinase activity |
| 22 | PLP2 | proteolipid protein 2 | acylglycerol lipase activity; identical protein binding; lipase activity; phospholipase activity | lipid catabolic process; lipid metabolic process; oxylipin biosynthetic process |
| 23 | PLPP2 | phospholipid phosphatase 2 | phospholipid dephosphorylation;signal transduction;phospholipid metabolic process | phosphatase activity |
| 24 | PLPP4 | phospholipid phosphatase 4 | diacylglycerol diphosphate phosphatase activity; identical protein binding; phosphatase activity; phosphatidate phosphatase activity | Fc-gamma receptor signaling pathway involved in phagocytosis; phospholipid dephosphorylation; phospholipid metabolic process |
| 25 | PLSCR1 | phospholipid scramblase 1 | CD4 receptor binding; enzyme binding; nuclease activity; phospholipid scramblase activity; SH3 domain binding | phosphatidylserine biosynthetic process; plasma membrane phospholipid scrambling; positive regulation of gene expression; regulation of Fc receptor mediated stimulatory signaling pathway |
| 26 | PLSCR4 | phospholipid scramblase 4 | CD4 receptor binding; enzyme binding; epidermal growth factor receptor binding; phospholipid scramblase activity; SH3 domain binding | phosphatidylserine biosynthetic process; plasma membrane phospholipid scrambling; positive regulation of gene expression; regulation of Fc receptor mediated stimulatory signaling pathway; response to interferon-beta |
| 27 | PLTP | phospholipid transfer protein | protein-containing complex subunit organization;phospholipid transport;regulation of biological process;amide transport | phosphatidylcholine binding; amide binding; phosphatidylcholine transporter activity |
| 28 | SOAT1 | Sterol O-acyltransferase 1 | cholesterol metabolic process;cholesterol transport;lipid modification | cholesterol binding; O-acyltransferase activity; carbohydrate derivative binding; purine nucleotide binding; anion binding;amide binding |
| 29 | STARD13 | StAR related lipid transfer domain containing 13 | regulation of Rho protein signal transduction;Rho protein signal transduction | GTPase activity; GTPase activator activity |
| 30 | STARD7 | StAR related lipid transfer domain containing 7 | [lipid binding](https://www.ebi.ac.uk/QuickGO/term/GO:0008289) | inflammatory response; myeloid dendritic cell activation |
